# PDI–trityl dyads as photogenerated molecular spin qubit candidates†

**DOI:** 10.1039/d3sc04375d

**Published:** 2023-09-22

**Authors:** Maximilian Mayländer, Kevin Kopp, Oliver Nolden, Michael Franz, Philipp Thielert, Andreas Vargas Jentzsch, Peter Gilch, Olav Schiemann, Sabine Richert

**Affiliations:** a Institute of Physical Chemistry, University of Freiburg Albertstraße 21 79104 Freiburg Germany sabine.richert@physchem.uni-freiburg.de; b Clausius Institute of Physical and Theoretical Chemistry, University of Bonn Wegelerstraße 12 53115 Bonn Germany; c Institute of Physical Chemistry, Heinrich Heine University Düsseldorf Universitätsstraße 1 40225 Düsseldorf Germany; d SAMS Research Group, Université de Strasbourg, CNRS, Institut Charles Sadron UPR 22 67000 Strasbourg France

## Abstract

Owing to their potential applications in the field of quantum information science, photogenerated organic triplet–radical conjugates have attracted an increasing amount of attention recently. Typically, these compounds are composed of a chromophore appended to a stable radical. After initialisation of the system by photoexcitation, a highly spin-polarised quartet state may be generated, which serves as a molecular spin qubit candidate. Here, we investigate three perylene diimide (PDI)-based chromophore–radical systems with different phenylene linkers and radical counterparts by both optical spectroscopy and transient electron paramagnetic resonance (EPR) techniques. Femtosecond transient absorption measurements demonstrate chromophore triplet state formation on a picosecond time scale for PDI–trityl dyads, while excited state deactivation is found to be slowed down considerably in a PDI–nitroxide analogue. The subsequent investigation of the coherent spin properties by transient EPR confirms quartet state formation by triplet–doublet spin mixing for all investigated dyads and the suitability of the two studied PDI–trityl dyads as spin qubit candidates. In particular, we show that using tetrathiaryl trityl as the radical counterpart, an intense spin polarisation is observed even at room temperature and quartet state coherence times of 3.0 μs can be achieved at 80 K, which represents a considerable improvement compared to previously studied systems.

## Abstract 2

Quartet states, generated after photoexcitation of chromophore–radical dyads, are promising for applications in quantum sensing. We demonstrate the potential of trityl radicals for improving their coherence properties and spectral addressability.

## Graphical abstract

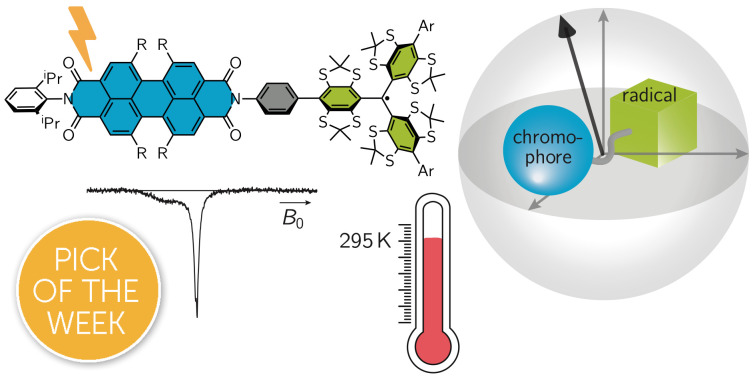

## 1 Introduction

Recent progress in the characterisation of the excited state properties of chromophore–radical systems, has revealed their potential use for a range of applications in the emerging field of molecular spintronics, including optoelectronic materials and quantum sensing devices.^1–4^ Among the various possible applications, the prospect to use molecular qubits for quantum sensing seems particularly attractive: compared to the frequently employed and well-established nitrogen vacancy (NV^−^) centres in diamond,^5,6^ molecular systems have the striking advantage that they are much smaller in size and that their structures can be defined with atomic precision and systematically modified by chemical synthesis.^7–12^ If the structure–property relationships in these materials are sufficiently well understood, it should thus be possible to optimise the molecular design and thereby tailor the magnetic properties to specific needs.

Photoexcited chromophore–radical systems share important characteristics with NV^−^ centres, notably the possibility of an optical activation and read-out, and can thus be seen as molecular NV^−^ centre surrogates. They are typically composed of three synthetic building blocks (*i.e.*, chromophore, linker, radical) that are highly modular. After photoexcitation of the chromophore to its first excited singlet state, the chromophore triplet state is rapidly formed by radical-enhanced intersystem crossing (EISC). Photoexcitation thus creates a second spin centre, the chromophore triplet state, which will subsequently interact with the appended stable radical. If the exchange coupling interaction between triplet state and radical (*J*_TR_) exceeds other magnetic interactions active in the spin system, quartet states may be formed by spin mixing and the system is then said to be in the so-called strong coupling regime.

In particular for chromophore–radical systems using perylene diimide (PDI) as the chromophore, such quartet states have previously been studied by transient pulse electron paramagnetic resonance (EPR) techniques.^2,4^ It has been found that these high-spin states exhibit properties that make them suitable as molecular spin qudits (*i.e.* qubits with more than two levels) for applications in quantum information science: they can be initialised by light in a pure spin state, are long lived, and are characterised by coherence times that are longer than typical pulse microwave-driven gate operation times, even at moderate temperatures of 80 K.

On the example of a PDI–nitroxide dyad, it has recently been shown that coherent spin manipulation is also possible at room temperature,^13^ illustrating that the main requirements for quantum sensing applications can in principle be fulfilled with such systems. However, a better understanding and further improvement of the coherence properties is desirable for an integration into functional spintronic devices.

Previous studies investigating the properties of photogenerated quartet states made use of 2,2,6,6-tetramethylpiperidinyloxyl (TEMPO)^2^ or 1,3-bisdiphenylen-2-phenylallyl (BDPA)^4^ radicals, whereby spin coherence times up to 2 μs were reported at 80 K. The data available thus far in the literature, suggests that there might exist a correlation between the coherence time of the radical and the phase memory time of the resulting quartet state.^13^ Consequently, we envisioned that the favourable coherence properties of tetrathiaryl trityl radicals, especially at elevated temperatures, could lead to an enhancement of the quartet state coherence times. Trityl radicals are frequently employed as spin labels^14–17^ and are characterised by a particularly narrow EPR line and slow spin relaxation.^18^

**Fig. 1:**
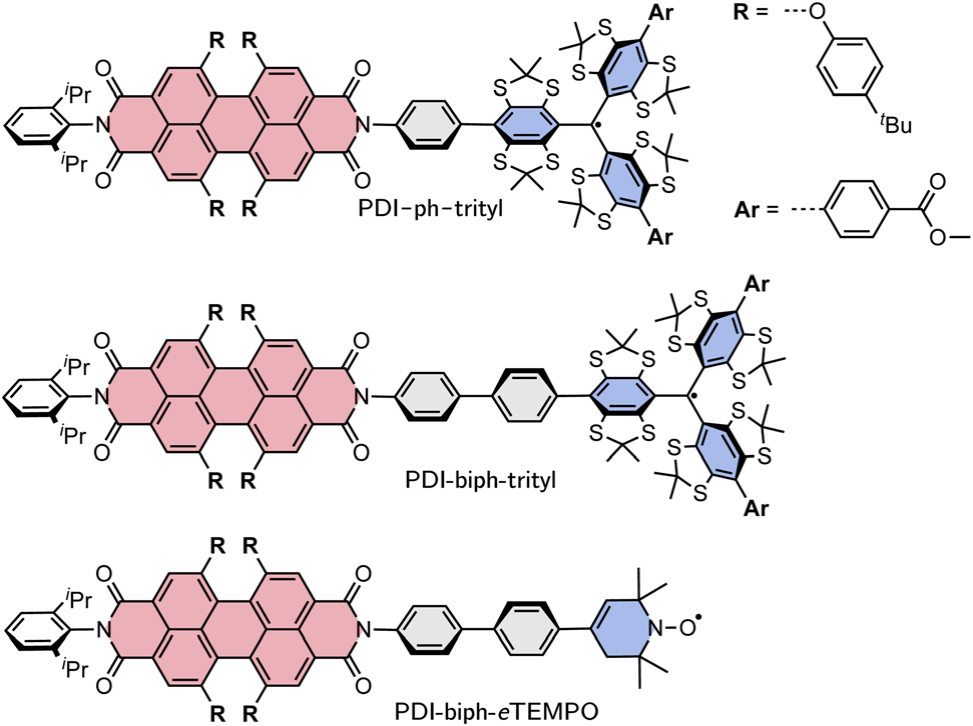
Structures of the three investigated PDI-based dyads: PDI-ph-trityl, PDI-biph-trityl, and PDI-biph-*e*TEMPO.

Here, we investigate two PDI–trityl dyads with different linkers (phenyl *vs.* biphenyl) and compare their photophysical behaviour with that of a third dyad with biphenyl linker where the trityl radical is replaced by a derivative of TEMPO with a double bond in the piperidine ring (*e*TEMPO, see Fig. 1).^19^ The structures are shown in Fig. 1 and are referred to as PDI-ph-trityl, PDI-biph-trityl, and PDI-biph-*e*TEMPO.

A detailed study of the optical properties using steady state and time-resolved spectroscopies reveals that the PDI triplet state is formed in all investigated dyads, albeit with different rate constants and yields. In the PDI–trityl dyads, excitation energy transfer could be identified as a potential loss channel limiting the triplet yield. Nevertheless, the triplet yield of these two dyads is significantly higher than that of PDI-biph-*e*TEMPO, resulting in the observation of intense spin-polarised quartet state signals in transient pulse EPR experiments in frozen solution. We show that this intense spin polarisation is preserved even at room temperature by characterising the magnetic properties and the time-evolution of the spin states both in isotropic liquid solution and in polymer films. Finally, a complete analysis of the coherent spin properties at 80 K demonstrates that a quartet state coherence time of 3 μs can be achieved with PDI-biph-trityl, which represents a significant improvement compared to previously studied systems and an important step towards our goal of identifying the factors that will allow us to optimise the performance of photogenerated multi-spin systems for applications in molecular spintronic devices.

## 2 Results and discussion

The synthesis and characterisation of the two PDI–trityl compounds as well as a characterisation of their dark state EPR properties will be reported in due course elsewhere, while the synthesis and characterisation of the PDI-biph-*e*TEMPO dyad is outlined in Section 1 of the ESI.†

### 2.1 UV-vis spectroscopy

The UV-vis absorption spectra of the three dyads in toluene solution at room temperature are shown in Fig. S1 in the ESI.† As also observed for most previously investigated chromophore–radical systems, it can be seen that the spectra of the dyads represent a superposition of the absorption spectra of the chromophore and radical precursors, indicative of weak electronic coupling between them. The PDI chromophore is characterised by three prominent absorption peaks at 579 nm, 537 nm, and 449 nm, while the absorption spectrum of the trityl radical has a prominent peak at 461 nm and a low-intensity absorption tail extending out to ∼900 nm.^3,20^

The absorption of the *e*TEMPO radical peaks at 458 nm in toluene and decays to baseline level at about 600 nm.^19^ However, since the peak molar absorption coefficient of the *e*TEMPO radical (21.2 M^−1^ cm^−1^)^19^ is very small as compared to that of PDI (3.1 × 10^4^ M^−1^ cm^−1^),^21^ the UV-vis spectrum of PDI-biph-*e*TEMPO is virtually identical to that of the PDI chromophore on its own. In contrast, the absorption peaks of the trityl radical are very prominent in the spectra of PDI-ph-trityl and PDI-biph-trityl, as the peak absorption coefficient of trityl is similar to that of PDI.

In the two PDI–trityl dyads, the strong fluorescence of the PDI chromophore in toluene solution at room temperature (*Φ*_F_ = 0.92)^21^ is considerably quenched, corresponding to fluorescence quantum yields of 0.2% and 4% for PDI-ph-trityl and PDI-biph-trityl, respectively. On the other hand, we find that the PDI-biph-*e*TEMPO dyad is still highly fluorescent under the same conditions with a quantum yield of 81%, pointing towards major differences in the excited state deactivation behaviour.

**Fig. 2:**
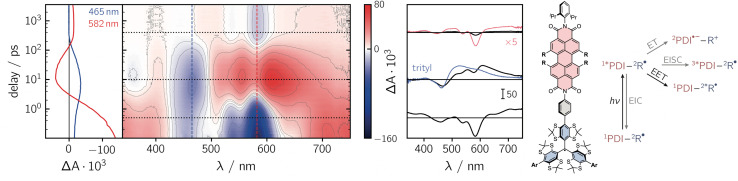
Contour plot of the fsTA data for PDI-ph-trityl dissolved in toluene solution at room temperature after photoexcitation at 535 nm. The red and blue colour coding in the contour plot represents positive and negative signals, respectively. The vertical coloured lines indicate the positions corresponding to the kinetic traces shown in the left panel, while the dotted horizontal lines indicate the time delays associated with the spectra shown on the right. The spectrum overlaid in blue is taken from the fsTA data recorded for an isolated trityl radical in ref. 20 and the spectrum overlaid in red represents a zoom (×5) into the data to illustrate the spectral shape. The general photoscheme to the right of the fsTA data shows the possible excited state deactivation pathways in covalently-linked chromophore–radical systems (neglecting any residual fluorescence). The numeric superscripts indicate the spin multiplicity. Abbreviations: R-radical, EISC-enhanced intersystem crossing, EIC-enhanced internal conversion, EET-excitation energy transfer, ET-electron transfer.

To get further insight into the mechanisms controlling the photo-induced kinetic processes in the three investigated compounds, the excited state deactivation kinetics after photoexcitation of PDI were studied using femtosecond UV-vis absorption spectroscopy (fsTA). The data obtained for PDI-ph-trityl in toluene solution at room temperature are shown in Fig. 2, while the corresponding data for PDI-biph-trityl and PDI-biph-*e*TEMPO are presented in the ESI (see Fig. S4 and S5†) together with a description of the experimental setup and parameters.

Immediately after photoexcitation, the fsTA spectrum of PDI-ph-trityl shows the typical signatures of the PDI excited singlet state as reported earlier.^21,22^ The intense ground state bleach signal between 400 and 600 nm overlaps with stimulated emission extending from ∼580 to 750 nm and excited state absorption signals covering almost the entire visible range. At wavelengths beyond 600 nm, the stimulated emission is overcompensated by the excited state absorption. These signals decay in only a couple of picoseconds, accompanied by a marked change in the spectral shape. After about ∼10 ps, the fsTA spectrum resembles that of the excited state of trityl, reported in ref. 20, indicative of excitation energy transfer (EET) taking place. The peak of the ground state bleach is shifted to ∼470 nm and new excited state absorption signals arise in the range from ∼500 to 700 nm. Superimposed on the excited state absorption of the trityl radical, a minor contribution from the ground state bleach of PDI can still be discerned. The signals attributed to the excited trityl radical are then found to decay completely within approximately 400 ps, leaving a low-intensity spectrum behind, that exhibits the known characteristics of the PDI excited triplet state.^21^

A qualitatively similar behaviour is observed for PDI-biph-trityl, while the fsTA spectrum of PDI-biph-*e*TEMPO shows almost no decay of the PDI excited singlet state within the time window accessible by fsTA measurements (see Fig. S4 and S5†).

To determine the time constants of the excited state deactivation, a global kinetic analysis of the fsTA data was carried out and the results, including the decay and species associated spectra, are presented and discussed in the ESI (Section 2.5 and Fig. S6 to S9).† The analysis revealed that the PDI excited singlet state decays with a time constant *τ*_S1_ of 2.5 ps in PDI-ph-trityl, while the decay is found to be slightly slower for PDI-biph-trityl (8.7 ps). In both cases, the excited state absorption of the trityl radical is then found to decay with a time constant of ∼90 ps, similar to that determined for an isolated trityl radical.^20^ The signal remaining after ∼450 ps, treated as a constant offset in the kinetic analysis, can be attributed to the PDI excited triplet state. The experimentally determined time constants *τ*_S1_ are in good agreement with time constants *τ*_FRET_ as predicted by Förster theory (see the ESI Section 2.2 and Table S1†), indicating that the depletion of the PDI singlet excitation is predominately due to Förster resonance energy transfer (FRET).

In the case of PDI-biph-*e*TEMPO, all signals in the fsTA spectrum, attributed to the excited singlet state of PDI, are found to decay collectively with a time constant in the nanosecond range. For the kinetic analysis, this time constant was set to the value of 5.2 ns, determined in a single photon timing experiment (see Fig. S2†).

For the two PDI–trityl compounds, the yield of triplet state formation can be estimated by comparing the amplitude of the ground state bleach immediately after photoexcitation (0.1 ps) and at a later time (∼450 ps) where PDI triplet state formation can be considered to be complete (*i.e.*, after complete decay of the excited state absorption signals of the trityl radical). Applying this procedure, we obtain triplet yields of 10% for PDI-ph-trityl and 12% for PDI-biph-trityl. These yields are in line with the predominance of the FRET quenching mentioned above.

The estimated triplet yields from fsTA measurements are also in excellent agreement with the results obtained when measuring the singlet oxygen quantum yield of the dyads as detailed in the ESI (see Section 2.6 and Fig. S10).† The measured singlet oxygen quantum yield amounts to 10% for both PDI-ph-trityl and PDI-biph-trityl, while we obtained a value of 4% for PDI-biph-*e*TEMPO. Table 1 provides an overview of the photophysical properties of all three dyads in toluene solution at room temperature that were discussed so far.^23^

**Table 1:** Overview of the photophysical properties in toluene at 295 K. *Φ*_F_: fluorescence quantum yield; *τ*_S1_: excited singlet state deactivation time constant of PDI; *Φ*_T_: triplet yield from fsTA; *Φ*_Δ_: singlet oxygen quantum yield

Compound	*Φ*_F_	*τ*_S1_/ps	*Φ*_T_	*Φ*_Δ_
PDI-ph-trityl	0.002	2.5	0.10	0.10
PDI-biph-trityl	0.04	8.7	0.12	0.10
PDI-biph-*e*TEMPO	0.81	5.2 × 10^3^	n.a.	0.04

### 2.2 Pulse EPR characterisation in frozen solution

To characterise the magnetic properties of the dyads, pulse EPR measurements were performed at the Q-band (34.0 GHz) in frozen toluene solution at 80 K. A detailed description of the sample preparation and experimental setup is provided in the ESI (Sections 3.1 and 3.2).†

**Fig. 3:**
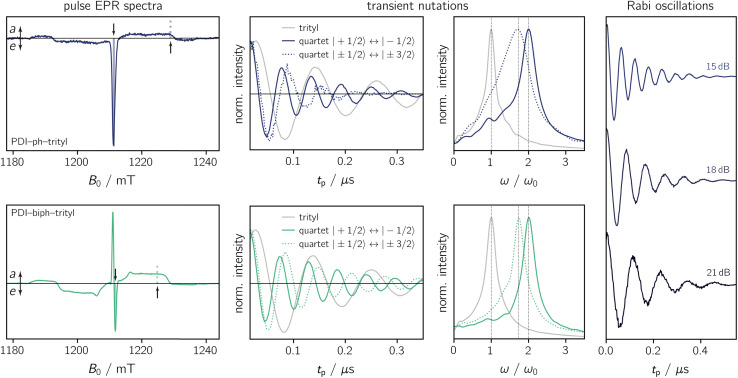
Pulse EPR data for PDI-ph-trityl and PDI-biph-trityl in frozen toluene solution at 80 K. Left: Field-swept echo-detected EPR spectra for PDI-ph-trityl (top) and PDI-biph-trityl (bottom) recorded after photoexcitation at 535 nm. The residual dark state signal was subtracted from the data. The solid and dashed vertical lines indicate the field positions where the transient nutation and Rabi oscillation measurements were performed. Centre: Transient nutation data and corresponding Fourier transform. The light-induced signals were referenced against the data recorded for the trityl radical in the dark (*ω*_0_). Right: Rabi oscillations measured at the field position corresponding to the central quartet line for PDI-ph-trityl at different microwave attenuations (as indicated).

The light-induced pulse EPR spectra of PDI-ph-trityl and PDI-biph-trityl in frozen toluene solution are shown in Fig. 3 (left), whereas the spectrum obtained for PDI-biph-*e*TEMPO is shown in the ESI (Fig. S19†). While intense signals are observed for both PDI–trityl dyads, the light-induced signal of PDI-biph-*e*TEMPO is very weak, in line with its small triplet yield. The following investigations will therefore focus on the two PDI–trityl compounds.

The spectrum of PDI-ph-trityl is characterised by a very intense, purely emissive, net polarisation and a weak *aeeaae* multiplet polarisation. In contrast, the net polarisation of PDI-biph-trityl shows an *ae* pattern and the intensity of the *aeeaae* multiplet polarisation is increased considerably compared to PDI-ph-trityl. To confirm the quartet nature of the light-induced signals, transient nutation experiments were carried out as shown in Fig. 3 (centre). Details regarding the experimental setup are provided in the ESI.†

According to the relation^24–26^
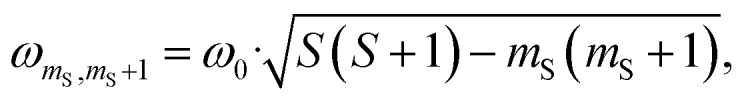
a frequency of 2*ω*_0_ would be expected for signals associated with the net polarisation of the quartet state, *i.e.* the |*m*_S_ = +1/2〉 ↔ |*m*_S_ = −1/2〉 transition, while a frequency of 
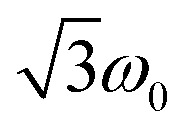
 is characteristic for any transitions between the |±1/2〉 and |±3/2〉 states (multiplet polarisation). The reference frequency *ω*_0_, of a species with pure doublet multiplicity, is obtained by performing the same experiment in the dark where only the radical signal can be detected. As shown in Fig. 3, the measured frequencies are in excellent agreement with those expected, leaving no doubt about the quartet nature of the observed signals.

**Fig. 4:**
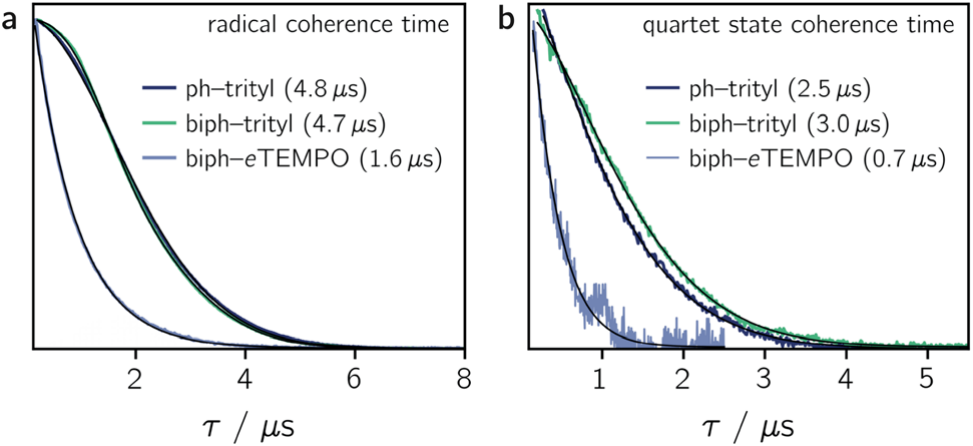
Spin echo decay for PDI-ph-trityl, PDI-biph-trityl, and PDI-biph-*e*TEMPO measured at the Q-band in frozen toluene solution at 80 K in the dark (a) and after photoexcitation at 535 nm (b). The spin coherence times (*T*_m_), obtained from a stretched exponential fit, are indicated. The coherence time of the quartet state was measured at a field position corresponding to the |*m*_S_ = +1/2〉 ↔ |*m*_S_ = −1/2〉 transition. Any residual dark state signal was suppressed during the quartet state *T*_m_ measurements by application of a pre-saturation pulse.

Fig. 4a shows the spin echo decay measured for the three dyads in the dark at a magnetic field position corresponding to the intensity maximum of the field-swept echo-detected EPR spectrum (see Fig. S18 and S19†). The fit of a stretched exponential function *V*_SE_ = exp(−(2*τ*/*T*_m_)^*β*^) to the data yielded the spin coherence times (*T*_m_) indicated in the figure. It can be seen that *T*_m_ of the trityl radical is roughly the same for PDI-ph-trityl and PDI-biph-trityl and amounts to 4.7 μs at 80 K with *β* values of 1.9 and 2.3, respectively, while that of *e*TEMPO is considerably shorter (1.6 μs, *β* = 1).

Having confirmed the favourable coherence properties of trityl radicals as compared to nitroxides, we wanted to explore, in a next step, whether the enhancement of the radical spin coherence time can be used as a strategy to improve the spin coherence properties of the photogenerated quartet states.

The spin coherence times measured at a field position corresponding to the net polarisation of the quartet state (|*m*_S_ = +1/2〉 ↔ |*m*_S_ = −1/2〉 transition, see the marked positions in Fig. 3) are compared in Fig. 4b. Focussing on the comparison between PDI-biph-trityl and PDI-biph-*e*TEMPO, the data illustrate very clearly that an enhancement of the radical coherence time goes along with a comparable enhancement of the coherence time of the corresponding photogenerated quartet state. Such a correlation has been suggested already in an earlier study comparing isotopically labelled PDI–TEMPO compounds,^13^ but additional experiments, such as these, involving different radicals and chromophores will be necessary in future to establish a clear relationship.

For PDI-ph-trityl, the feasibility of a coherent manipulation of the spin states was also demonstrated by performing a Rabi oscillation experiment. The data recorded for different microwave attenuations are shown in Fig. 3 (right). For microwave attenuations of 15, 18, and 21 dB (corresponding to microwave powers of 0.316, 0.159, and 0.0794 mW) we obtain Rabi frequencies of 17.2, 12.4, and 8.6 MHz, respectively, at 80 K. From this observed linear relationship between the Rabi frequency (*Ω*_R_) and the microwave *B*_1_ field, it can be concluded that the oscillations are of pure Rabi type and, consequently, that the studied spin qubit candidate can be placed in any arbitrary superposition state.^27^ Finally, the figure of merit *Ω*_M_ = 2*T*_m_*Ω*_R_ indicates that >80 single-qubit logic operations can be performed with PDI-ph-trityl at 80 K (*T*_m_ = 2.5 μs). For PDI-biph-trityl (*T*_m_ = 3.0 μs), this implies that at least 100 rotations should be feasible in any pulse microwave-driven experiment at 80 K.

### 2.3 Transient EPR spectroscopy at room temperature

**Fig. 5:**
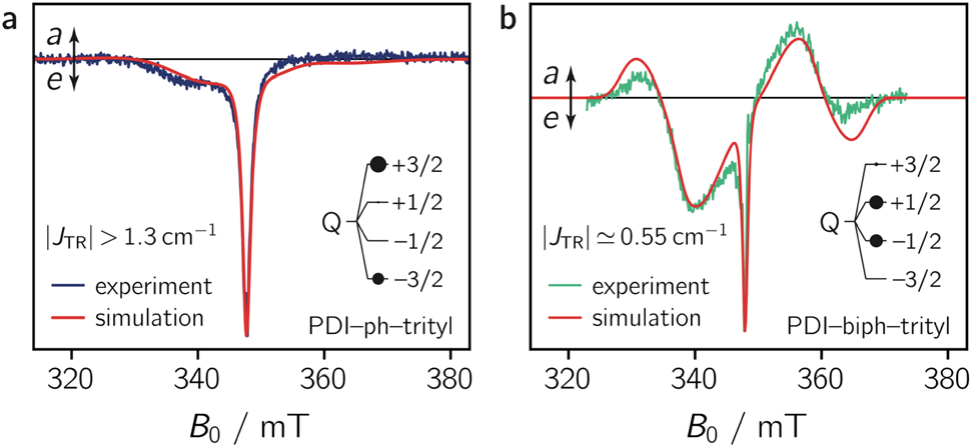
Room temperature trEPR spectra of PDI-ph-trityl (a) and PDI-biph-trityl (b) in toluene solution at 0.4 μs after laser excitation together with the best fit to the data assuming a quartet spin state. The relative quartet state sublevel populations, obtained from the simulations, are indicated in the inset by black circles.

The samples for the transient continuous wave (trEPR) measurements at room temperature were prepared as outlined in the ESI.† The experiments were performed at the X-band both in liquid toluene solution and in polymer (PMMA) films. The spectra obtained for the two PDI–trityl compounds in toluene solution are shown in Fig. 5, while the data for the film samples can be found in the ESI.†

Fig. 5 shows that the strong emissive net spin polarisation, observed for PDI-ph-trityl in isotropic frozen solution, is conserved at room temperature. While the multiplet polarisation of PDI-ph-trityl is found to be even less intense at room temperature than at 80 K, it is clearly present in the spectrum of PDI-biph-trityl in liquid solution. The latter spectrum shows a close resemblance to that in frozen solution, apart from the fact that the individual features are less well resolved, potentially caused by a larger conformational spread.

As can be seen in Fig. S13 in the ESI,† the shape of the trEPR spectra of the PDI–trityl compounds is found to change markedly as a function of time after photoexcitation, pointing towards a contribution of several species. As expected, the multiplet polarisation of the quartet state decays faster than the net polarisation.^28,29^ In addition, a close look at the central emissive feature in the spectrum of PDI-ph-trityl at early times (<1.5 μs) reveals a double-peak structure, whereby the intensities of the two peaks show a different decay behaviour. The right-most peak, associated with an isotropic *g* value of ∼2.0025, can be attributed to the polarised trityl ground state, while the other peak, at lower field, is associated with a *g* value of ∼2.0045 and might thus be assigned to the excited doublet state according to *g*_D_1__ = 1/3 (4 *g*_T_ − *g*_R_).^30^ The signal assigned to the polarised D_0_ state is characterised by a slower decay as compared to the D_1_ state and still remains emissive while the net polarisation decays and changes its phase. Eventually, the D_0_ state signal disappears, leaving only a weak absorptive signal behind that takes the shape of the net polarisation of the quartet state and persists for several microseconds. Fig. S14 in the ESI† shows that a similar observation is also made for PDI-biph-trityl, supporting the assignment of the two peaks. However, at the X-band, a *g*-value difference of 0.001 only corresponds to a Δ*B*_0_ of 0.175 mT, meaning that any D_0_ or D_1_ state signals will necessarily overlap with the broad emissive net polarisation of the quartet state centred at *g* = 2.0035, in line with *g*_Q_ = 1/3 (2*g*_T_ + *g*_R_). To confirm the observation of a polarised D_0_ and a D_1_ state in addition to the quartet state, additional trEPR measurements at higher microwave frequencies would be required.

A qualitatively similar time-behaviour is also observed when embedding the two PDI–trityl molecules in PMMA films (see Fig. S15 and S16†). A close comparison of the spectral shapes reveals that the linewidths are slightly increased in PMMA films and notably, that the signal decay is slowed down considerably (see also Fig. S17†). The partial immobilisation of the molecules in PMMA films thus has the advantage that spin relaxation is slowed down to an extent that even pulse EPR measurements may become feasible.^13^ In addition, it goes along with an improved photostability of the samples as compared to the measurements in liquid solution.

To characterise the magnetic parameters of the formed quartet states, spectral simulations of the room temperature solution spectra of PDI-ph-trityl and PDI-biph-trityl were carried out using EasySpin.^31,32^ The best fits to the data are shown together with the spectra in Fig. 5. The spectra were simulated assuming a coupled two-spin system with *S*_1_ = 1 and *S*_2_ = 1/2. To reduce the number of variable parameters, the triplet state and radical precursors were characterised separately (see the ESI, Section 3.3†), and their magnetic parameters (*g*_R_, *g*_T_, *D*_T_) were kept fixed in the simulation of the dyad spectra. For an accurate determination of the trityl **g** tensor, a room temperature X-band continuous wave EPR spectrum was fit together with the corresponding pulse Q-band spectrum recorded at 80 K. This global analysis yielded ***g***_R_ = [2.003 2.002] as illustrated in Fig. S12 in the ESI.† The magnetic parameters of the PDI triplet state precursor were already determined in a previous study and amount to *g*_T_ = 2.0040, *D*_T_ = 970 MHz, and *E*_T_ = −185 MHz.^13^ Further details on the simulation procedure and a list of all simulation parameters are provided in the ESI.†

As a result of the simulations, it is found that the magnetic interactions in PDI-ph-trityl place the system clearly within the strong coupling regime, where the magnitude of *J*_TR_ has no influence any more on the spectral shape. In contrast, the best fit for the spectrum of PDI-biph-trityl is obtained when assuming an exchange coupling in the upper intermediate coupling regime, where *J*_TR_ can still be determined experimentally as it influences the width and zero-crossing points of the outer spectral features (multiplet polarisation). Consequently, for PDI-ph-trityl, only a lower limit for |*J*_TR_| can be given experimentally, which amounts to ∼1.3 cm^−1^ (=40 GHz). In the case of PDI-biph-trityl, we obtain the best agreement between experimental data and simulation for *J*_TR_ ≃ −0.55 cm^−1^ (=16.6 GHz).

Quantum chemical calculations of the excited state exchange coupling parameters, as illustrated in detail in the ESI (see Section 4.3 and Fig. S24),† reveal that the coupling is ferromagnetic^33^ (*E*_Q_ < *E*_D_1__) in both cases, as previously also observed experimentally for a CuTPP-ph-trityl compound.^34^ Although these calculations are known to underestimate the magnitude of the exchange coupling substantially,^35^ the sign of *J*_TR_ and the computed trends in the exchange coupling parameters are generally found to be reliable. For the two PDI–trityl dyads, calculations predict a decrease in |*J*_TR_| by roughly an order of magnitude (factor of ∼40) when an additional phenyl spacer is introduced in PDI-biph-trityl (see Table S4 in the ESI†). Relying on the experimentally determined value of *J*_TR_ ≃ −0.55 cm^−1^ for PDI-biph-trityl and the computed change in the exchange interaction by a factor of 40, this suggests a *J*_TR_ value of roughly −22 cm^−1^ for PDI-ph-trityl.

The numerical simulations shown in Fig. 5 further indicate that the observed differences in the spectral shape between PDI-ph-trityl and PDI-biph-trityl (notably the relative intensity of the multiplet polarisation) can be explained by a clear difference in the relative populations of the four quartet state sublevels, in addition to the large difference in the magnitude of |*J*_TR_|. While the quartet state ±|3/2〉 levels are overpopulated in PDI-ph-trityl, the population is shifted to the ±|1/2〉 levels in the case of PDI-biph-trityl. The latter observation suggests a difference in the mechanism responsible for doublet–quartet spin mixing in the two dyads.

Spin mixing, *i.e.* the reversible transition between the D_1_ and quartet states, can either be mediated by dipolar interactions or spin–orbit coupling.^1,28,36–39^ As proposed recently,^19^ it is likely that the different magnitudes of |*J*_TR_| in the two dyads result in differences in the efficiencies and relative importance of the possible doublet–quartet mixing mechanisms. Dipolar-induced mixing^1,36,38^ becomes less efficient with increasing |*J*_TR_| as the rate has an inverse dependence on the energy gap between the trip-doublet and trip-quartet states. On the other hand, SOC-induced mixing^1,28,36^ has no clear dependence on |*J*_TR_| and might therefore become dominant in cases where dipolar-induced mixing is inefficient.

### 2.4 Design considerations and comparison to similar systems

The triplet yield of ∼10% achieved for both PDI–trityl dyads compares well with that of a similar, PDI-based, system making use of BDPA as the radical counterpart.^4^ Based on the relatively large distance (∼2 nm) between chromophore and radical in the PDI–trityl dyads, direct EISC would be expected to be rather slow.^19^ In addition, we observe an *aeeaae* multiplet polarisation pattern in the transient EPR spectra, as also observed for a PDI–TEMPO dyad where this was suggested to arise from electron transfer followed by rapid charge recombination to the chromophore triplet state (so-called “inverted kinetics”).^21^ Although no direct experimental proof is available, inverted kinetics cannot be excluded as an additional deactivation pathway of the chromophore exciting singlet state (see the ESI, Section 2.3,† for energetic considerations) and might be responsible for the non-negligible triplet yield observed here despite EET taking place.

Given that the absorption spectrum of the trityl radical covers the entire visible range, EET can only be avoided with trityls if chromophore and radical are connected in a way so that their transition dipole moments are perpendicular. Alternatively, a chromophore with a small radiative rate constant could be used. In contrast, to avoid EET when working with BDPA or nitroxide radicals, it should be sufficient to select a chromophore with a fluorescence onset >580 nm.^3^

Compared to nitroxides, trityl radicals are characterised by improved coherence properties which translates to an increase in the coherence times of the corresponding quartet states as shown in this study. While, for a PDI–TEMPO and a PDI–BDPA compound, quartet state *T*_m_ values of ∼2 μs were measured in frozen toluene at 80 K,^4,13,21^ PDI-biph-trityl exhibits a substantially enhanced coherence time of ∼3 μs.

In more weakly-coupled triplet–radical systems, the narrow spectral line of the trityl radical could also be very useful for polarisation transfer experiments. Compared to BDPA radicals, the EPR linewidth is reduced by roughly a factor of three: a Δ*B*_pp_ of 0.056 mT was measured for trityl (see Fig. S11†), while, for BDPA, the corresponding value amounts to 0.17 mT.^4^ This translates to pulse EPR linewidths (FWHM) of 0.3 mT and 0.8 mT, for trityl and BDPA, respectively.^40^ On the other hand, the linewidths of any nitroxide radicals are significantly larger due to a considerable hyperfine coupling contribution. The large number of coupling nuclei has the additional disadvantage that it favours spin decoherence.

Finally, for the storage of spin information, electron spin lattice relaxation times (*T*_1_) are of significant interest. In this context, trityl radicals show again a clear advantage over nitroxides: while *T*_1_ values of 2 ms have been measured here for trityl radicals in frozen toluene solution at 80 K, the corresponding value for TEMPO only amounts to 0.2 ms.^2^ Among the different types of frequently used stable radicals, BDPA shows by far the slowest spin lattice relaxation with ∼40 ms at 80 K.^40^ However, the *T*_m_ of BDPA is inferior as compared to both nitroxides and trityls, which makes the use of trityl radicals overall most appealing.

## 3 Conclusions

Trityl radicals, known for their narrow EPR lines and improved spin relaxation properties as compared to nitroxides, have been employed here to demonstrate a link between the spin coherence time of the radical precursor and that of the photogenerated quartet state formed by spin mixing in covalently-linked triplet–radical systems. Using tetrathiaryl trityl as the radical counterpart in PDI–radical systems, the coherence time *T*_m_ of the photogenerated quartet states could be significantly enhanced as compared to previous studies making use of different radicals such as TEMPO and BDPA.^2,4,13^ The coherence time of the radical precursor could thus be identified here as an important handle to optimise the coherence times of photogenerated quartet states, which is expected to be crucial for their application as spin qudits in the field of quantum sensing.

For the two investigated PDI–trityl dyads, significant triplet yields of ∼10% were measured and resulted in the observation of a strong emissive spin polarisation of the quartet |+1/2〉 ↔ |−1/2〉 transition. The intense spin polarisation was shown to be preserved even at room temperature and the spin polarisation lifetime can be tuned by embedding the system in different matrices as demonstrated here by using polymer films. In case of PDI-ph-trityl, almost pure net emissive spin polarisation is observed and since the central quartet line is fairly narrow, the quartet state has the potential to be employed as a polarising agent in spin polarisation transfer experiments for the hyperpolarisation of nuclear spins, in analogy to previous work making use of photoexcited triplet electrons for DNP.^41^

Numerical simulations of the transient EPR spectra of PDI-ph-trityl and PDI-biph-trityl, supported by quantum chemical calculations, revealed major differences in the magnitude of the exchange interaction |*J*_TR_| and the relative quartet state sublevel populations, likely resulting from differences in the efficiencies and relative importance of the possible doublet–quartet mixing mechanisms. As suggested in an earlier study,^19^ the magnitude of |*J*_TR_| thus appears to play a role in determining the mechanism responsible for doublet–quartet spin mixing. Comparing the two EPR spectra, it seems that this change in mechanism mainly influences the relative intensities of the net and multiplet polarisation, whereby a large value of |*J*_TR_| results in almost pure net polarisation. It remains, however, to be verified whether this observation also holds true for other systems of this kind.

Finally, we can conclude that the favourable spin relaxation properties of trityl radicals are certainly an asset that will help to advance the application of chromophore–radical systems in the areas of quantum sensing and hyperpolarisation. However, to increase the potential of any trityl-based triplet–radical systems further, future work will need to concentrate on identifying a modified design that (i) preserves the stability, narrow EPR lines, and long coherence times of trityl radicals, but (ii) reduces their tendency to act as an energy acceptor in excitation energy transfer reactions.

## Data availability

The data supporting the findings of this study are available within the article and in the ESI.†

## Author contributions

Spectroscopic characterisation of the compounds, EPR data acquisition and analysis, fsTA data analysis, draft editing and data visualisation M. M.; Synthesis and characterisation of the PDI–trityl compounds K. K.; FsTA data acquisition and analysis, draft editing O. N.; quantum chemical calculations and analysis of excited state exchange couplings, draft editing M. F.; preparation of the polymer film samples, EPR data acquisition, draft editing P. T.; synthesis and characterisation of the PDI-*e*TEMPO dyad, draft editing A. V. J.; supervision of the fsTA experiments, funding acquisition, draft editing P. G.; supervision of the synthesis, funding acquisition, draft editing O. S.; conceptualisation, project administration, funding acquisition, supervision of the optical and EPR experiments, original draft writing, data visualisation S. R.

## Conflicts of interest

There are no conflicts to declare.

## Supplementary Material

SC-014-D3SC04375D-s001
