# Supplementary material for: PDI–trityl dyads as photogenerated molecular spin qubit candidates
Source: Chem Sci. 2023 Sep 22;14(39):10727–35. doi: 10.1039/d3sc04375d (PMC10566479; doi:10.1039/d3sc04375d)
Supplement: SC-014-D3SC04375D-s001 [file SC-014-D3SC04375D-s001.pdf]

Electronic Supplementary Material (ESI) for Chemical Science.
This journal is © The Royal Society of Chemistry 2023

Supporting Information for

PDI−trityl dyads as photogenerated molecular spin qubit candidates

Maximilian Mayländer,[1] Kevin Kopp,[2] Oliver Nolden,[3] Michael Franz,[1] Philipp Thielert,[1] Andreas Vargas Jentzsch,[4] Peter Gilch,[3] Olav Schiemann,[2] Sabine Richert [1]*

[1] *Institute of Physical Chemistry, University of Freiburg, Albertstraße 21, 79104 Freiburg, Germany*

[2] *Institute of Physical and Theoretical Chemistry, University of Bonn, Wegelerstraße 12, 53115 Bonn, Germany*

[3] *Institute of Physical Chemistry, Heinrich Heine University Düsseldorf, Universitätsstraße 1, 40225 Düsseldorf, Germany*

[4] *SAMS Research Group, Université de Strasbourg, CNRS, Institut Charles Sadron UPR 22, 67000 Strasbourg, France*

* E-mail: sabine.richert@physchem.uni-freiburg.de

Table of Contents

1 Synthesis of PDI−biph−*e*TEMPO **S1**
- 1.1 General methods . . . S1
- 1.2 Synthetic protocols . . . S1

2 Optical spectroscopic characterisation **S2**
- 2.1 UV-vis spectroscopy . . . S2
- 2.2 Calculation of the Förster resonance energy transfer rate constants . . . S3
- 2.3 Calculation of the driving forces for electron transfer . . . S4
- 2.4 Femtosecond TA data acquisition . . . S5
- 2.5 Additional fsTA data and global kinetic analysis . . . S6
- 2.6 Determination of the triplet yields . . . S9

3 EPR characterisation **S11**
- 3.1 EPR sample preparation . . . S11
- 3.2 EPR setup and parameters . . . S11
- 3.3 EPR characterisation of the radical and triplet precursors . . . S13
- 3.4 Characterisation of the coupled systems by trEPR . . . S14
- 3.5 Simulation of the trEPR spectra . . . S17
- 3.6 Additional pulse EPR data . . . S18

4 Quantum chemical calculations **S19**
4.1 Optimisation of the structures . . . S19
4.2 Triplet state energy of PDI . . . S19
4.3 Excited state exchange couplings . . . S20

5 NMR spectra **S22**

6 HRMS data **S23**

List of Figures

S1 UV-vis spectra of the investigated PDI dyads . . . S2
S2 Fluorescence decay trace of PDI−biph−*e*TEMPO . . . S3
S3 Spectral overlap of chromophore emission and radical absorption . . . S3
S4 Contour plot of the fsTA data for PDI−biph−trityl . . . S6
S5 Contour plot of the fsTA data for PDI−biph−*e*TEMPO . . . S7
S6 Decay associated spectra for the PDI−trityl compounds . . . S7
S7 Species associated spectra for PDI−ph−trityl . . . S8
S8 Species associated spectra for PDI−biph−trityl . . . S8
S9 Species associated spectra for PDI−biph−*e*TEMPO . . . S9
S10 Determination of the singlet oxygen quantum yield . . . S10
S11 Comparison of the cw EPR data for all investigated dyads . . . S13
S12 Dark state EPR parameters of PDI−ph−trityl . . . S13
S13 Room temperature trEPR spectra of PDI−ph−trityl in toluene at different delays . . . S14
S14 Room temperature trEPR spectra of PDI−biph−trityl in toluene at different delays . . . S15
S15 Comparison of the room temperature trEPR spectra in films . . . S15
S16 Room temperature trEPR spectra in films at different delays . . . S15
S17 Comparison of the trEPR data of PDI−ph−trityl recorded under different conditions . . . S16
S18 Pulse EPR spectra of the PDI−trityl compounds . . . S18
S19 Pulse EPR spectra of PDI−biph−*e*TEMPO at 80 K . . . S18
S20 Spin polarisation lifetime for the PDI−trityl dyads at 80 K . . . S18
S21 Optimised structures of the PDI−trityl dyads . . . S19
S22 Optimised structure of the PDI−biph−*e*TEMPO dyad . . . S19
S23 Optimised structures of the PDI S_0 and T_1 states . . . S19
S24 Active orbitals and their individual exchange interactions . . . S21
S25 ^{1}H NMR spectrum of PDI−biph−*e*TEMPO . . . S22
S26 HRMS-ESI analysis for PDI−biph−*e*TEMPO . . . S23

1 Synthesis of PDI–biph–*e*TEMPO

1.1 General methods

All reactions were performed under an argon atmosphere unless otherwise indicated. All reagents and solvents were purchased at the highest commercial quality and used without further purification unless otherwise noted. Thin layer chromatography was performed with TLC silica on aluminium foil (Silica gel/UV254, Aldrich). Irradiation using a Bioblock VL-4C UV-lamp (6 W, 254 nm and/or 365 nm) was used as well as suitable TLC stains for visualisation. Preparative thin layer chromatography (PTLC) was performed using commercial silica gels plates (Merck Z740216, 60 Å pore size). ^{1}H NMR spectra were recorded on a Bruker Avance III HD 400 MHz spectrometer equipped with a BBFO probe at 298 K. The spectra were internally referenced to the residual proton solvent signal. For ^{1}H NMR the chemical shifts are given in ppm. Coupling constants *J* are listed in Hz. Standard abbreviations indicating multiplicity were used as follows: s = singlet, d = doublet, dd = doublet of doublets, t = triplet, q = quartet, quintet, m = multiplet, br = broad signal. Ultra performance liquid chromatography coupled to mass spectrometry (UPLC-MS) was carried out on a Waters Acquity UPLC-SQD apparatus equipped with a PDA detector (190–500 nm, 80 Hz), using a reverse phase column (Waters, BEH C18 1.7 μm, 2.1 mm $\times$ 50 mm), and the MassLynx 4.1 – XP software with a gradient (water-acetonitrile + 0.1 % formic acid) as eluent. High-resolution mass spectra (HRMS) were recorded on a Thermo Fisher Scientific Exactive mass spectrometer with an orbitrap analyser using either atmospheric pressure chemical ionisation (APCI) or electrospray ionisation (ESI).

1.2 Synthetic protocols

The synthetic procedure to prepare I-*e*TEMPO has been reported previously,[1] and the synthesis of precursor PDI–biph–Bpin will be reported in due time as part of a different publication that includes also the syntheses of PDI–ph–trityl and PDI–biph–trityl. Nevertheless, the corresponding ^{1}H NMR spectrum is reported in a double panel (Figure S25) showing the expected changes following the preparation of PDI–biph–*e*TEMPO. While a complete structural elucidation of radical compounds by NMR is not possible in chromophore–radical systems like those reported here, it is often possible to clearly identify the features distant from the radical unit.

Compound PDI–biph–*e*TEMPO

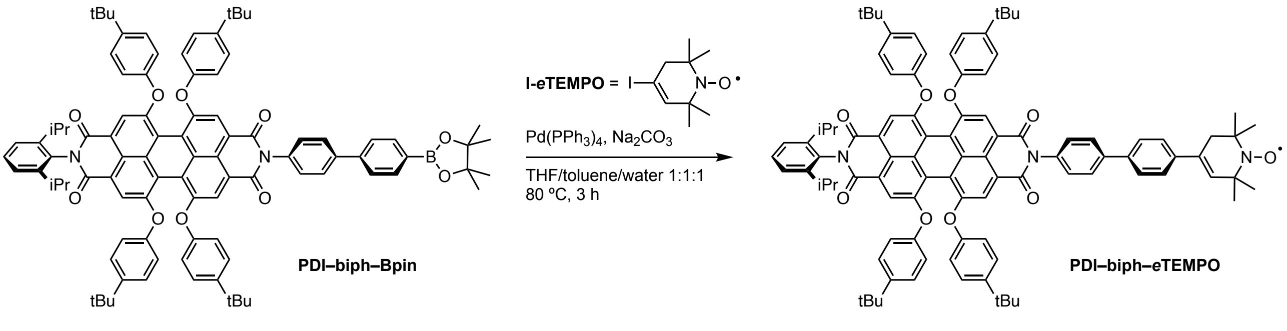

In a round-bottom flask (50 mL), PDI–biph–Bpin, (1 eq., 10 mg, 7 μmol), I-*e*TEMPO (1.5 eq., 3.0 mg, 10.6 μmol) and sodium carbonate (3.9 eq., 3.0 mg, 28 μmol) were dissolved in a mixture of water/THF/toluene (1:1:1 - 18 mL). $Pd(PPh_3)_4$ (0.05 eq., 0.4 mg, 0.4 μmol) was added and the reaction

mixture was stirred at 80 °C for 3 h (monitoring by UPLC). After the reaction was completed, a saturated solution of Na_2CO_3 (15 mL) was added and the mixture was extracted with dichloromethane (3×50 mL). The combined organic extracts were dried over Na_2SO_4 and filtered. The solvent was removed under reduced pressure and the residue was purified by PTLC (SiO_2, DCM) to yield PDI−biph−*e*TEMPO (9 mg, 6.2 μmol, 88.4%) as a deep purple solid. R_f (DCM 2%(v/v) acetone) ∼ 0.4.

^{1}H NMR (400 MHz, $CDCl_3$) δ 8.27 (s, 2H), 8.23 (s, 2H), 7.76 (s br, 2H), 7.39 (t, J = 7.7 Hz, 2H), 7.28−7.16 (m, 8H), 6.84 (dd, J = 17.0, 8.5 Hz, 8H), 2.68 (quintet, J = 6.6 Hz, 2H), 1.32−1.16 (m, 36H), 1.10 (d, J = 6.8 Hz, 12H).

HRMS-ESI calculated for $C_{97}H_{96}N_3O_9^{\bullet}$ $[M+H]^+$: 1447.7219, found: 1447.7227.

2 Optical spectroscopic characterisation

2.1 UV-vis spectroscopy

Steady-state absorption measurements of the samples in toluene were carried out on a Shimadzu UV-1601 UV-vis spectrometer. Figure S1 shows the normalised UV-vis spectra of the three investigated dyads.

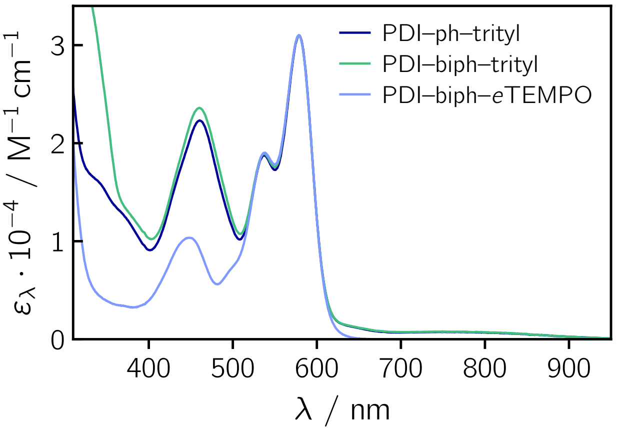

Figure S1: UV-vis spectra of the three investigated PDI-based dyads. The spectra have been scaled to match the molar absorption coefficient of PDI of $3.1 \cdot 10^4\ M^{-1}cm^{-1}$ at 579 nm. [2]

For the fluorescence measurements, the samples were diluted substantially, corresponding to absorbances < 0.1 at the excitation wavelength. Steady-state fluorescence spectra were recorded on a FluoroMax-4 fluorimeter from Horiba. The raw spectra were corrected for the spectral sensitivity of the instrument and fluctuations of the excitation light source.

Fluorescence quantum yields were determined using a C11347 absolute photoluminescence quantum yield spectrometer from Hamamatsu Photonics K.K., Japan. The same solutions as prepared for the fluorescence measurements were used. An excitation wavelength of 530 nm was chosen and the analysis was performed using the tools provided with the data acquisition software.

Fluorescence lifetime measurements were carried out using a FluoTime 100 fluorescence lifetime spectrometer from Picoquant GmbH, Germany. The samples were excited at 470 nm and the scattering light from the excitation source was cut off with the help of a long-pass filter placed in the detection path. The instrument response function was collected (without any filters) using a solution of LUDOX® (colloidal silica) in distilled water. To obtain the fluorescence decay times, iterative re-convolution of the instrument response function with a monoexponential decay function was performed in MATLAB. The model

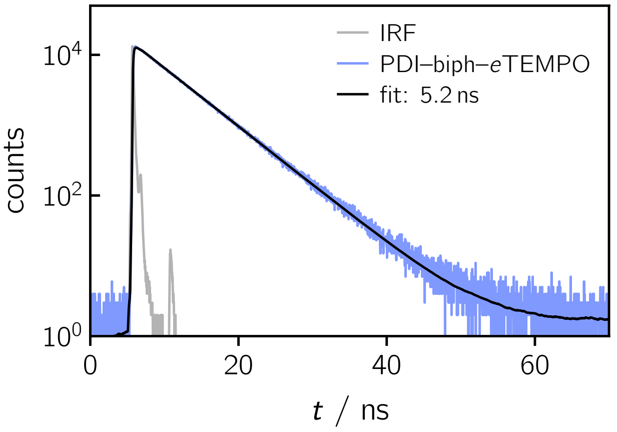

Figure S2: Fluorescence decay of PDI−biph−*e*TEMPO measured by single photon timing and best fit to the experimental data.

decay function was fit to the experimental data using a least-squares fitting approach (minimisation of the residuals using a built-in trust-region-reflective algorithm). The data obtained for PDI−biph−*e*TEMPO are shown in Figure S2.

2.2 Calculation of the Förster resonance energy transfer rate constants

Since Förster-type excitation energy transfer (FRET) between PDI and trityl is very likely and has previously been observed,[3] we calculated the theoretically expected EET rate constants for the investigated dyads.

As can be seen from the formulae for the calculation of the FRET rate constants below, i.e. Equations (S1) to (S3), the efficiency of this process will depend primarily on (i) the spectral overlap of chromophore emission and radical absorption and, (ii) the relative orientation of the transition dipole moments of chromophore and radical (κ^2), in addition to the distance r_{DA} between the donor and acceptor moieties. A visualisation of the spectral overlap is shown in Figure S3. While it is significant in the case of trityl, it can be seen that there is almost no spectral overlap between the PDI emission and the absorption of the *e*TEMPO radical.

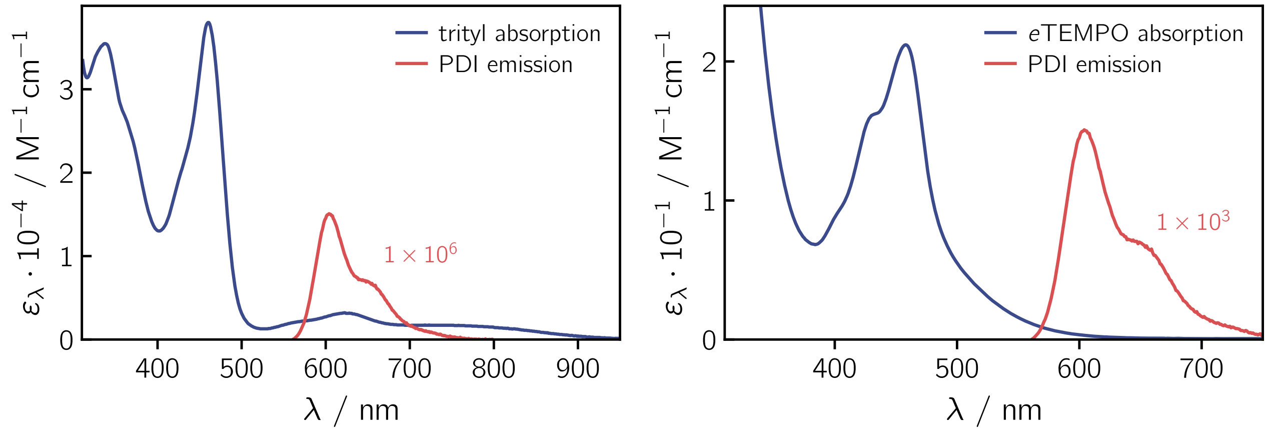

Figure S3: UV-vis absorption spectrum of the trityl radical (*left*) and *e*TEMPO radical (*right*) together with the fluorescence emission spectrum of PDI for an illustration of the spectral overlap.

The transition dipole moment (TDM) of the PDI chromophore lies along the long axis of the molecule, which corresponds to the chromophore−radical bonding axis. Since the TDM of trityl is also roughly alined with the bonding axis,[4] a κ^2 value of four can be assumed. As shown previously,[1] the TDM of

the *e*TEMPO radical is perpendicular to the bonding axis, so that $\kappa^2 = 0$. Considering also the poor spectral overlap, FRET can most likely be excluded for PDI−biph−*e*TEMPO.

For the calculation of the FRET rate constants k_{FRET} for the PDI−trityl dyads, the molar absorption coefficient of the trityl radical was taken to amount to $3.8 \cdot 10^4\,\mathrm{M^{-1}cm^{-1}}$ at its absorption maximum of 460 nm.[3] The fluorescence quantum yield and lifetime of the PDI chromophore were taken from reference 2, while the center-to-center distances, r_{DA}, were taken from DFT models of the structures (vide infra) and amount to 1.59 nm for PDI−ph−trityl and 2.02 nm for PDI−biph−trityl, respectively. An overview of the results and parameters is provided in Table S1.

Table S1: Overview of the results from the calculation of the Förster radius and FRET time constant for the investigated structures. The following parameters were used: $r_{\mathrm{DA}} = 1.59$ nm for PDI−ph−trityl and 2.02 nm for PDI−biph−trityl, $n = 1.496$ (toluene), $\tau^{\mathrm{D}}_{\mathrm{F,0}} = 5.9$ ns, $\Phi^{\mathrm{D}}_{\mathrm{F,0}} = 0.92$, $\varepsilon^{\mathrm{A}} = 3.8 \cdot 10^4\,\mathrm{M^{-1}cm^{-1}}$ for trityl.

compound	κ^2	R_0 / nm	τ_{FRET} / ps
PDI−ph−trityl	4 (collinear)	5.45	3.6
PDI−biph−trityl	4 (collinear)	5.45	15

The Förster radius R_0 (obtained in nm) can be calculated from[5]

$$(R_0)^6 = 8.785 \cdot 10^{-11} \frac{\Phi^{\mathrm{D}}_{\mathrm{F,0}}\,\kappa^2}{n^4} \int I^{\mathrm{D}}_{\mathrm{F}}(\lambda)\,\varepsilon^{\mathrm{A}}(\lambda)\,\lambda^4\,\mathrm{d}\lambda \tag{S1}$$

with

$$\int I^{\mathrm{D}}_{\mathrm{F}}(\lambda)\,\mathrm{d}\lambda = 1 \tag{S2}$$

where $\Phi^{\mathrm{D}}_{\mathrm{F,0}}$ and $I^{\mathrm{D}}_{\mathrm{F}}$ are the fluorescence quantum yield and fluorescence intensity of the donor, ε^{A} is the molar absorption coefficient (in $\mathrm{M^{-1}cm^{-1}}$) of the acceptor and n the refractive index of the medium. The orientation factor κ^2 accounts for the relative orientation of the two transition dipole moment vectors (emission of donor and absorption of acceptor) with respect to the axis connecting the FRET pair.

The energy transfer rate constant is then given as

$$\tau^{-1}_{\mathrm{FRET}} = k_{\mathrm{FRET}} = \frac{1}{\tau^{\mathrm{D}}_{\mathrm{F,0}}} \left(\frac{R_0}{r_{\mathrm{DA}}}\right)^6 \tag{S3}$$

where $\tau^{\mathrm{D}}_{\mathrm{F,0}}$ is the fluorescence lifetime of the donor in the absence of any quenchers and r_{DA} is the center-to-center distance (point dipole) between donor and acceptor.

2.3 Calculation of the driving forces for electron transfer

The calculations of the driving forces $-\Delta G_0$ for charge separation (CS) and charge recombination (CR) were performed assuming the validity of the following equations:[6]

$$-\Delta G_{0,\,\mathrm{CS}} = -\Delta G_{0,\,\mathrm{IP}} + E_{00} \tag{S4}$$

$$-\Delta G_{0,\,\mathrm{CR}} = \Delta G_{0,\,\mathrm{IP}} \tag{S5}$$

$$\Delta G_{0,\,\mathrm{IP}} = e\,[E_{\mathrm{ox}}(\mathrm{D}) - E_{\mathrm{red}}(\mathrm{A})] + C + S \tag{S6}$$

where the subscript IP stands for ion pair, E_{00} is the energy of the first excited singlet state and $E_{\mathrm{ox}}(\mathrm{D})$ and $E_{\mathrm{red}}(\mathrm{A})$ are the oxidation potentials of the electron donor and reduction potentials of the electron acceptor, respectively. The terms C and S represent the coulomb and solvent correction terms, defined as

$$C = -\frac{e^2}{4\pi\varepsilon_0\varepsilon_r r_{\mathrm{ee}}} \qquad S = \frac{e^2}{8\pi\varepsilon_0}\left(\frac{1}{r_{\mathrm{D}}} + \frac{1}{r_{\mathrm{A}}}\right)\left(\frac{1}{\varepsilon_r} - \frac{1}{\varepsilon_{r,\,\mathrm{ref}}}\right) \tag{S7}$$

where r_{ee}, ε_r, ε_0, $\varepsilon_{r,\,\mathrm{ref}}$ and r_i are the edge-to-edge distance between the reaction partners, the relative solvent permittivity, the vacuum permittivity, the relative permittivity of the solvent used to determine the redox potentials and the Van-der-Waals radii, respectively.

The edge-to-edge distances for electron transfer and Van-der-Waals radii for donor (trityl) and acceptor (PDI) were estimated from DFT models of the structures (vide infra). They were taken as $r_{\mathrm{ee}} = 8.6$ Å for PDI−ph−trityl, $r_{\mathrm{ee}} = 13$ Å for PDI−biph−trityl, $r_{\mathrm{D}} = 10$ Å, and $r_{\mathrm{A}} = 6.3$ Å.

The standard electrode potential of the trityl radical ($E_{\mathrm{trityl^+/trityl}}$) is 0.61 V vs. SCE (in H_2O),[7] while the standard electrode potential of the PDI acceptor ($E_{\mathrm{PDI/PDI^-}}$ vs. SCE) amounts to −0.67 V (in CH_2Cl_2, $\varepsilon_r = 9$).[8] The solvent toluene, used for the spectroscopic measurements, has a relative dielectric constant of $\varepsilon_r = 2.4$ at room temperature.[9]

E_{00} for PDI (corresponding to E_{S1}) is calculated from the crossing point of the absorption and fluorescence spectra and amounts to 2.11 eV (588 nm). The energy of the PDI triplet state was estimated from quantum chemical calculations (vide infra) and amounts to $E_{\mathrm{T1}} = 1.30$ eV. The energy of the charge transfer state generated by electron transfer from trityl to PDI, E_{CT}, corresponds to $-\Delta G_{0,\,\mathrm{CR}}$ and was calculated using the parameters given above. It amounts to roughly 1.34 eV for PDI−ph−trityl, and 1.57 eV for PDI−biph−trityl, which corresponds to values for $-\Delta G_{0,\,\mathrm{CS}}$ of 0.77 eV and 0.54 eV, respectively. Finally, the excited state energy of the trityl radical, $E_{\mathrm{R*}}$, can be estimated from its UV-vis absorption spectrum and amounts to roughly 900 nm, i.e. 1.38 eV.

According to these calculations, electron transfer could thus be feasible in toluene. The absence of any ion signatures in fsTA experiments might be explained by inverted kinetics as suggested also earlier for a PDI–TEMPO dyad.[2] From a purely energetic point of view, recombination to the PDI triplet state should be possible from both the charge separated state and the excited doublet state of the radical, as they lie energetically above the excited triplet state of the PDI chromophore.

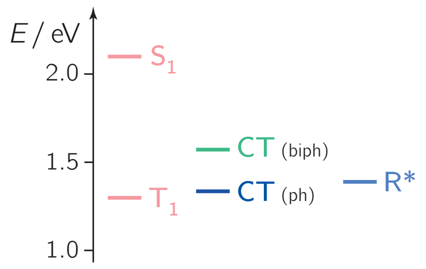

2.4 Femtosecond TA data acquisition

The setup has been described in detail elsewhere.[10–13] In brief, a Ti:Sapphire amplified laser system (Coherent Libra) with a repetition rate of 1 kHz, a pulse duration of 100 fs, and a wavelength of 800 nm was used as the pulse source. Part of its output was used to pump a TOPAS-White non-collinear optical parametric amplifier tuned to deliver pulses peaking at 530 nm. The pump power at the sample position amounted to 1 mW (i.e., 1 μJ/pulse). For probing (330−740 nm), a supercontinuum was generated in a CaF_2 plate. The pump beam diameter at the sample was 160 μm (FWHM), while the diameter of the probe beam was 100 μm. The relative polarisation of pump and probe beams was set to the magic angle and the instrument response time was ∼180 fs (FWHM).

Femtosecond transient UV-vis absorption (fsTA) spectra were recorded at 139 different time delays, where 50 linear time steps between −1 and 1 ps were followed by linear steps on a logarithmic scale up to roughly 4 ns. A total of 2000 spectra were acquired per time point and averaged over four successive scans. The chopper in the probe beam path was set to a frequency of 500 Hz, while that in the pump path was set to 250 Hz. For every time point, four different sets of data were collected: (i) only the white light (probe) reaches the sample, (ii) pump and probe both blocked, (iii) pump and probe both reach the sample, (iv) only the pump reaches the sample. Signals (i) and (iii) are used for the calculation of ΔA, while signals (ii) and (iv) are needed to account for dark signals and pump light scattering. The chirp of the white light was measured in a separate (optical Kerr effect, OKE) experiment and accounted for in the processing of the TA data. In addition, solvent spectra were recorded separately under identical conditions as the samples and subtracted from the sample data following the procedure detailed in reference 14.

The TA data were analysed using lab-written MATLAB routines. After subtraction of the suitably scaled solvent background, the data were chirp-corrected by interpolation in the time domain using a function of the form $f(t_D) = p_1 + \frac{p_2}{t_D^2} + \frac{p_3}{t_D^4}$, where $f(t_D)$ is the instrumental delay time and the parameters p_n are obtained by fitting the temporal peaks of the OKE response as a function of wavelength.

For the room temperature femtosecond TA experiments the samples were prepared in toluene solutions with an absorbance between 0.3 and 0.4 at the excitation wavelength of 530 nm in a 1 mm cuvette. During the experiments, the sample solutions (∼ 2 mL) were flown continuously. In addition, UV-vis spectra were taken before and after the measurements to verify the sample absorbances and confirm the absence of sample degradation during the measurement.

2.5 Additional fsTA data and global kinetic analysis

The contour plots of the fsTA data recorded for PDI−biph−trityl and PDI−biph−*e*TEMPO are shown in Figures S4 and S5. Compared to the data for PDI−ph−trityl discussed in the main part, it can be seen that the photophysical behaviour of PDI−biph−trityl is qualitatively similar, only that the excitation energy transfer is somewhat slower. In contrast, neither excitation energy or electron transfer seem to occur in PDI−biph−*e*TEMPO, resulting in a comparatively slow deactivation of the excited singlet state of PDI (5.2 ns, see Figure S2), as might be expected for the rather large distance between chromophore and radical of about 1.9 nm.[1]

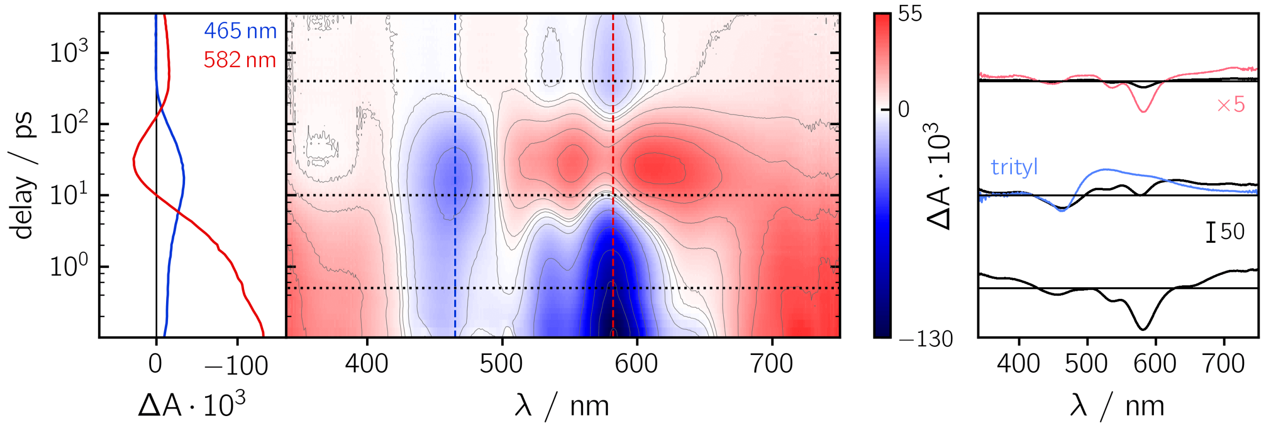

Figure S4: Contour plot of the fsTA data for PDI−biph−trityl recorded in toluene solution at room temperature after photoexcitation at 535 nm.

To determine the time constants of the excited state deactivation processes, a global kinetic analysis

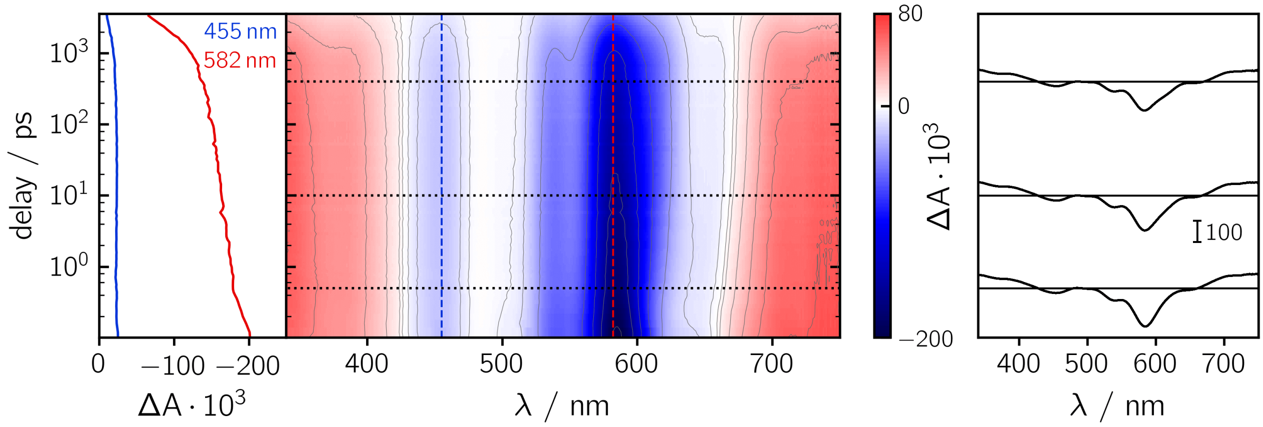

Figure S5: Contour plot of the fsTA data for PDI−biph−*e*TEMPO recorded in toluene solution at room temperature after photoexcitation at 535 nm.

of the fsTA data was carried out for all compounds. In a first step, the number of time constants required to reproduce the experimental data satisfactorily was determined by computation of the decay associated spectra (DAS).[15] Here, a multi-exponential function convoluted with the instrument response function (IRF) served as the trial function. In the case of PDI−ph−trityl, two time constants and an offset were required, while an additional sub-picosecond time constant needed to be included in the case of PDI−biph−trityl. The DAS obtained for the PDI−trityl compounds are shown in Figure S6.

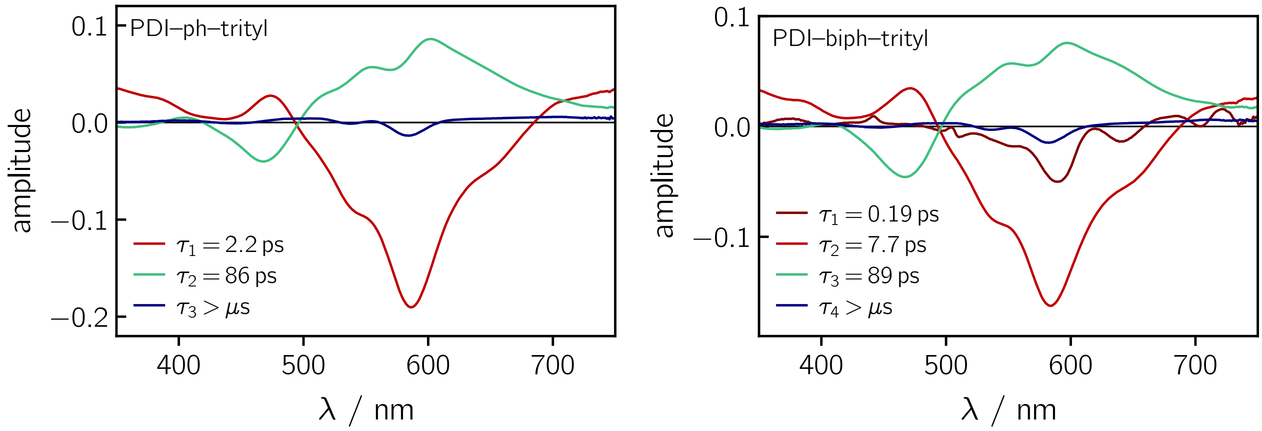

Figure S6: Decay associated spectra and time constants obtained from a global kinetic analysis of the fsTA data of PDI−ph−trityl (*left*) and PDI−biph−trityl (*right*) in toluene.

The two time constants can be assigned to the deactivation of the excited singlet state of PDI, mostly by excitation energy transfer, and the decay of the excited state absorption (ESA) of the trityl radical. The remaining signals (offset) represent the triplet/quartet state, the lifetime of which cannot be assessed by fsTA. In the case of PDI−biph−trityl, the assignment is the same, with the only difference that an additional time constant of ∼0.2 ps was necessary to reproduce the experimental data at early times. This time constant likely accounts for vibrational relaxation within the PDI excited singlet state. In both cases, the ESA of the trityl radical decays with a time constant of ∼90 ps. However, the decay of the PDI excited singlet state by EET is considerably faster in PDI−ph−trityl (2.2 ps) as compared to PDI−biph−trityl (7.7 ps) due to a larger distance between chromophore and radical, in agreement with calculations of the Förster energy transfer rate constants (see Subsection 2.2).

In a second step, species associated spectra (SAS) were computed assuming a branched model of the form:

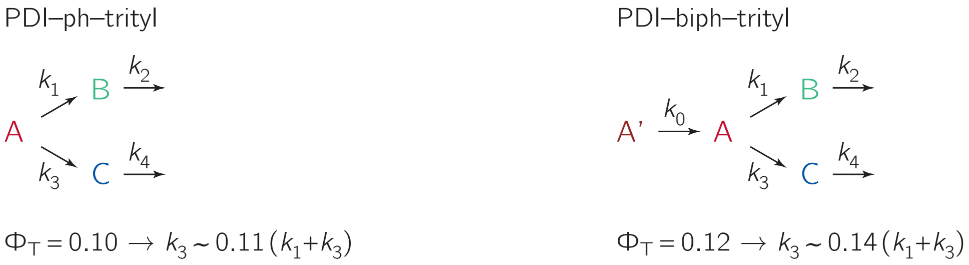

for the two PDI–trityl compounds and a sequential model (A' $\xrightarrow{k_0}$ A $\xrightarrow{k_1}$) for PDI–biph–*e*TEMPO. The species A', A, B, and C are assigned to the vibrationally hot excited singlet state of PDI, the excited singlet state of PDI, the excited trityl radical, and the PDI triplet state, respectively. The branching ratio was adapted based on the experimentally determined triplet yields of 10 % for PDI–ph–trityl and 12 % for PDI–biph–trityl.

The SAS obtained by plotting the contributions (i.e. amplitudes) of the individual time constants as a function of wavelength and assuming the validity of the chosen models are shown in Figures S7 to S9. Table S2 gives a summary of all time constants obtained from the kinetic analysis.

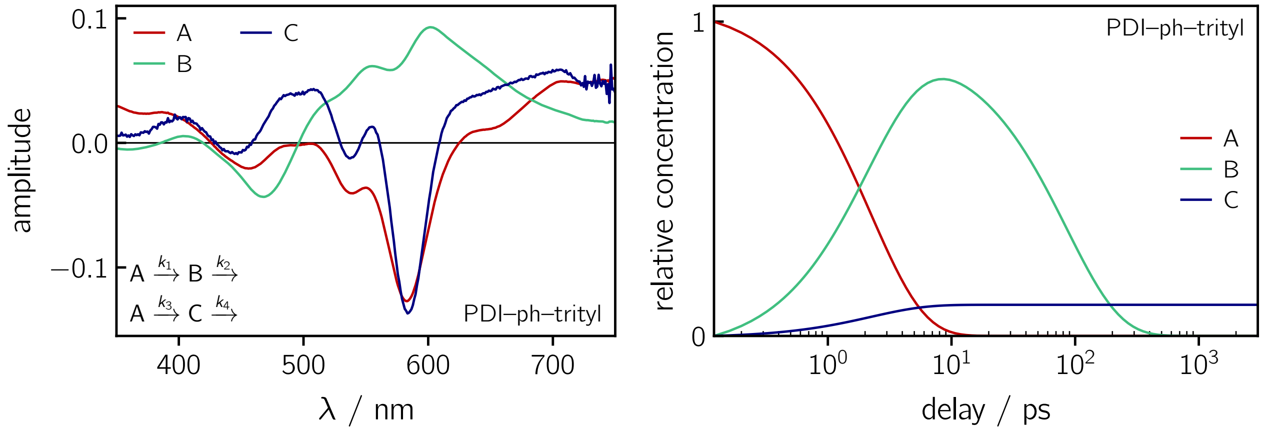

Figure S7: Species associated spectra obtained from a global kinetic analysis of the fsTA data of PDI–ph–trityl in toluene (*left*) and corresponding kinetics (*right*).

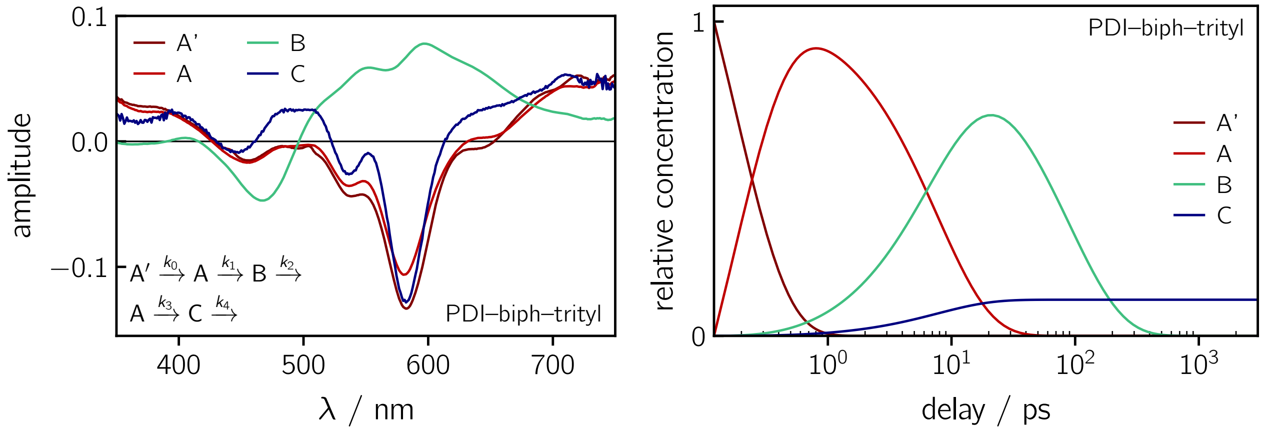

Figure S8: Species associated spectra obtained from a global kinetic analysis of the fsTA data of PDI–biph–trityl in toluene (*left*) and corresponding kinetics (*right*).

The time constants obtained for the formation and decay of the different species (elementary time constants) assuming a branched kinetic model are slightly different as compared to the lifetimes from the model-free analysis above. In addition, when incorporating the known triplet yield as an additional constraint, the time constant of triplet state formation can be calculated.

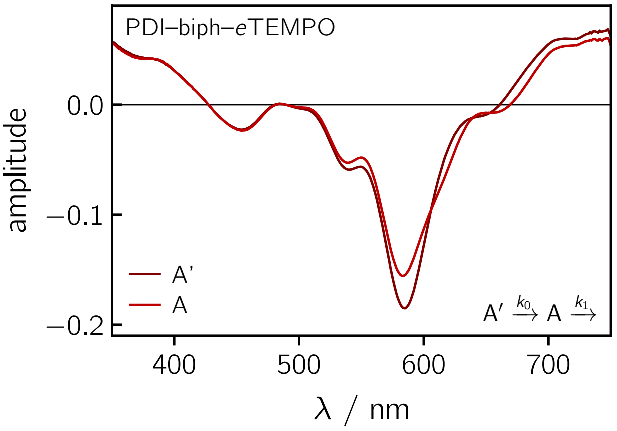

Figure S9: Species associated spectra obtained from a global kinetic analysis of the fsTA data of PDI–biph–*e*TEMPO in toluene. The excited singlet state lifetime was measured by single photon timing and fixed to the measured value of 5.2 ns.

In the case of PDI–biph–*e*TEMPO, triplet/quartet state formation cannot be monitored by fsTA since the kinetics are too slow. Presumably, this suggests that neither electron transfer or excitation energy transfer play a role here for excited state deactivation. The lifetime of the excited singlet state of PDI was measured by single photon timing and fixed to the determined value of 5.2 ns for the kinetic analysis.

Table S2: Time constants and triplet yields obtained from a global kinetic analysis (SAS) of the fsTA data of PDI–ph–trityl, PDI–biph–trityl, and PDI–biph–*e*TEMPO in toluene solution at room temperature. A red, green and blue colour coding is used for the time constants representing (i) the S_1 state deactivation of PDI, (ii) the decay of the excited state absorption of the trityl radical, and (iii) PDI triplet state formation, respectively.

compound	τ_1 / ps	τ_2 / ps	τ_3 / ps	τ_4 / ps	τ_5 / ps	Φ_T
PDI–ph–trityl	**2.5**	86	22	$> 10^6$	–	0.10
PDI–biph–trityl	0.18	**8.7**	89	64	$> 10^6$	0.12
PDI–biph–*e*TEMPO	5.2	$\mathbf{5.2 \cdot 10^3}$	–	–	–	N/A

2.6 Determination of the triplet yields

The triplet yield of the dyads can either be estimated from (i) measurements of the singlet oxygen quantum yield or by (ii) careful analysis of the time-dependence of the ground state bleach amplitude in fsTA experiments (cf. reference 2).

For the singlet oxygen quantum yield measurements, all samples were prepared in toluene solutions with matched absorbances of 0.1 (pathlength 10 mm) at the excitation wavelength of 510 nm. A Continuum Horizon OPO pumped by the third harmonic of a Continuum Surelite laser operated at 10 Hz was used for photoexcitation, whereby the laser intensity was adjusted to amount to 0.5 mJ at the sample using a combination of a half-waveplate and a Glan-Taylor polariser (spot size ∼5 mm, pulse length ∼5 ns). The samples (∼ 2.5 mL) were contained in quartz cuvettes with a pathlength of 10 mm inside a 3D-printed sample holder that can accommodate a cuvette-sized magnetic stirrer. During the measurements, the air saturated solutions were continuously stirred at a speed of 400 RPM to replenish the solution with oxygen.

Singlet oxygen is generated by reaction of molecular triplet states with triplet oxygen dissolved in the solution. The IR light emitted by $^1O_2$ was collected at 90° using a collimating lens and a back mirror both with a focal length of 50 mm. It passes an interference filter from Spectrogon adapted to the wavelength of 1270 nm before the signal is detected by an IR photomultiplier (Hamamatsu H10330-45) and recorded using a transient digitiser. A total of 256 averages were collected for each sample. The quenching of singlet oxygen is followed by monitoring the transient signal of $^1O_2$ and the intensity maximum of the signal after laser excitation allows the determination of the singlet oxygen quantum yield by comparison to the behaviour of a known system measured under identical conditions. Under the applied experimental conditions, it is assumed that triplet quenching by oxygen is complete, meaning that every quenching event generates singlet oxygen.

The data were referenced against PDI_{4sg}–TEMPO investigated in reference 2 with a known triplet state formation quantum yield of 50 %.

Figure S10 shows the transient singlet oxygen signal obtained for the three PDI-based dyads and the reference compound PDI_{4sg}–TEMPO[2] after photoexcitation at 550 nm. A narrow spike, originating from scattered excitation light, was observed at time zero. To avoid any effects of this spike on the intensity readings used for the determination of the singlet oxygen quantum yields, only the decay beyond 0.5 μs was analysed. To calculate the yields given in the main part the relative intensities at 1.0 μs were used.

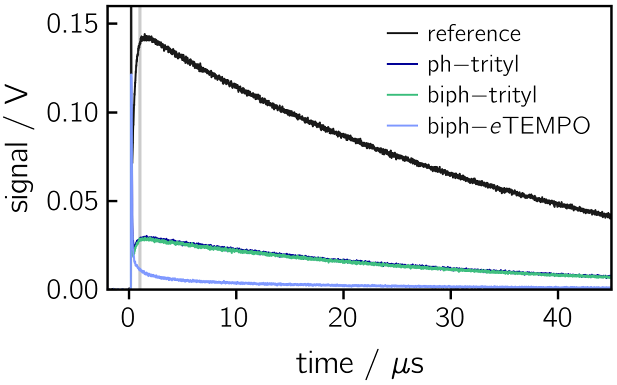

Figure S10: Measurement of the singlet oxygen signal as a function of time for the three investigated dyads and the PDI_{4sg}–TEMPO reference compound (cf. reference 2) with a known triplet yield of 50 %. The grey vertical line indicates the position where the intensities were read off for the determination of the singlet oxygen quantum yields.

For the cases of PDI–ph–trityl and PDI–biph–trityl, where the deactivation of the chromophore excited singlet state is observed in only a couple of picoseconds, triplet formation can also be monitored well by fsTA. By analysis of the amplitude of the ground state bleach directly after photoexcitation and at a later time, where triplet formation can be considered to be complete, the triplet yield can be estimated as also explained in more detail in the Supporting Information of reference 2.

The triplet yield of PDI–ph–trityl and PDI–biph–trityl was determined from the corresponding fsTA data by comparing the amplitude of the PDI ground state bleach directly after photoexcitation and at ~400 ps, where the excited state absorption of the trityl radical has decayed completely and the only remaining signals can be attributed to the PDI triplet state. In both cases, a value of approximately 10–11 % was obtained, which is in excellent agreement with the results of the singlet oxygen quantum yield measurements.

3 EPR characterisation

3.1 EPR sample preparation

EPR samples in toluene solution. For the transient EPR measurements in isotropic frozen solution, the samples were prepared with an absorbance of roughly 0.3 at the excitation wavelength of 535 nm, measured in a 2 mm cuvette. The samples in toluene were then transferred into quartz EPR tubes with an outer diameter of 1.6 mm (inner diameter of 1 mm) for measurements at the Q-band. The solutions were rapidly frozen in liquid nitrogen before insertion into the EPR resonator for the measurement.

The toluene samples used for room temperature X-band measurements were transferred into quartz EPR tubes with an outer diameter of 3.8 mm (inner diameter of 3 mm), degassed on a Schlenk line using the freeze-thaw technique and flame sealed.

EPR samples in polymer films. The polymer films were prepared using a 100 mg/mL stock solution of PMMA ($\sim 50-150\,\mu$m beads, MW 35,000; Thermo Fisher Scientific Inc.) in toluene as well as a sample stock solution in toluene with a concentration of 2−4 mg/mL. To dissolve PMMA and reach the desired concentration, the solution needed to be stirred for >48 h. For the preparation of the EPR samples, the sample and polymer stock solutions were mixed at a volumetric ratio of 1:1.

For trEPR measurements at the X-band, 11 μL of the analyte/PMMA solution were spread onto small quartz substrates (15 mm×2.6 mm×0.5 mm) by drop-casting and the sample was left to dry for 30 minutes while being uncovered. The drop-casting procedure was repeated on the backside of the substrate before insertion into an X-band EPR tube. The thin films feature an absorbance of roughly 1.3 at the excitation wavelength of 535 nm.

3.2 EPR setup and parameters

Continuous wave EPR spectroscopy. The spectra were recorded at the X-band (9.75 GHz) at room temperature on a Bruker EMXnano benchtop EPR spectrometer using quartz EPR tubes with an outer diameter of 3.8 mm (inner diameter of 3 mm). The modulation frequency was set to 100 kHz and the modulation amplitude to 0.1 mT at a microwave power of 1 mW (20 dB). The recorded, background-corrected, spectra were frequency-corrected to 9.75 GHz and field-corrected using a carbon fibre standard with $g = 2.002644$.[16]

Transient EPR spectroscopy. Transient pulse EPR measurements were performed at Q-band frequencies (34.0 GHz) on a Bruker ELEXSYS E580 spectrometer equipped with a Bruker EN45107D2 resonator. During the measurement, the sample was kept at a constant temperature of 80 K using an Oxford Instruments nitrogen gas-flow cryostat (CF 935). The samples were excited through the top of the sample holder with depolarised light at 535 nm using an optical fibre with a diameter of 0.8 mm. The excitation energy was ∼0.5 mJ at a repetition rate of 50 Hz (pulse duration ∼5 ns).

TrEPR measurements on film samples and liquid solutions were performed at the X-band as detailed below. For X-band EPR measurements, the samples were excited directly with depolarised laser light through the optical window of the cryostat and resonator (without an optical fibre). Compared to the Q-band measurements, slightly higher excitation energies of ∼1 mJ were used.

Transient continuous wave EPR. The spectra were acquired in direct detection mode with a video amplifier bandwidth of 20 MHz using the built-in transient recorder and a microwave power of 1.5 mW (20 dB). Any positive signals thus corresponds to an absorptive transition (a) and any negative signals to an emissive (e)

one. The transient recorder was used in AC-AFC mode and the transient signal fed through a low-noise voltage preamplifier (Stanford Research Systems SR 560) with a bandpass filter of 3 kHz−1 MHz before entering the detection circuitry. Typically, for every magnetic field value, a time trace with 4096 points was recorded using a time base of 4 ns. After data acquisition, the 2D spectra were baseline-corrected in both dimensions using a lab-written MATLAB routine.

The spectra shown in the figures have been averaged over a time window from 0.2 μs to 1 μs after laser excitation unless otherwise stated. All EPR spectra were frequency-corrected to either 9.75 GHz (X-band) or 34.0 GHz (Q-band) and field-corrected using a carbon fibre standard with $g = 2.002644$.[16]

Transient pulse EPR. Echo-detected field-swept EPR spectra at the Q-band were recorded using the sequence $h\nu - \text{DAF} - \pi/2 - \tau - \pi - \tau -$ echo with DAF $= 9\,\mu$s at 80 K (DAF $\equiv$ delay after flash), $\tau = 180$ ns, and a π-pulse length of 32 ns. Unless otherwise stated, a two-step phase cycle was applied in all pulse EPR experiments. The same pulse lengths were also used for the determination of T_m. To record T_m of the quartet state, a pre-saturation pulse was applied to avoid any contributions of the radical signal to the spin relaxation decay of the quartet state.

For measurements of the polarisation lifetime, a Hahn echo detection sequence was used and the position of the laser pulse with respect to the echo detection sequence was varied by a gradual variation of the DAF. The pulse sequences used are shown below.

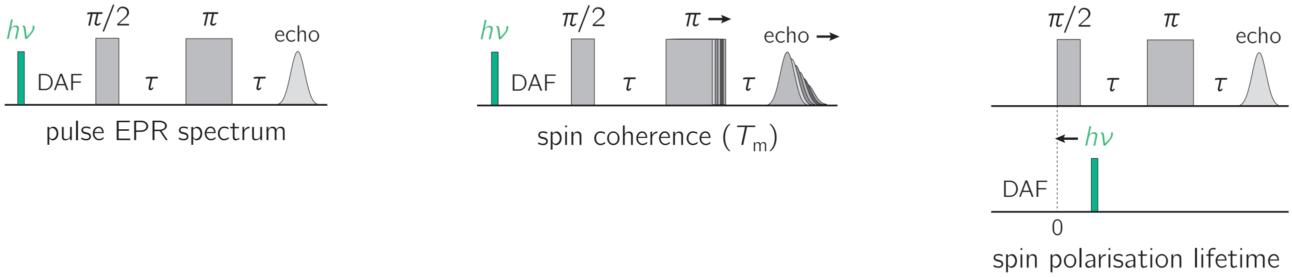

Transient nutation measurements used the sequence $h\nu - \text{DAF} - \xi - \tau - \pi - \tau -$ echo where the flip angle ξ was gradually increased by increasing the corresponding microwave pulse length in steps of 2 ns, starting at 16 ns ($\pi/2$). At specific magnetic field positions within the region of the quartet spectrum, the integrated echo intensity was then recorded as a function of this pulse length. The data were background corrected using a polynomial background function. The (cross-term averaged) Fourier transform was then calculated after dead-time reconstruction, windowing using a Hamming window, and zero filling to 2048 data points. The frequency spectra were normalised by division of the frequency axis by the reference frequency obtained for the same sample in the dark (doublet multiplicity). Whenever the dark state contribution to the transient signal at the chosen field position was still significant, a pre-saturation pulse was applied to limit any such contributions. This also applies to the transient Rabi oscillation experiments described hereafter.

Transient Rabi oscillation measurements used the sequence $h\nu - \text{DAF} - t_p - T - \pi/2 - \tau - \pi - \tau -$ echo where the pulse length t_p was gradually increased in steps of 2 ns, starting at 0 ns. At the chosen magnetic field position within the region of the quartet spectrum, the integrated echo intensity was then recorded as a function of this pulse length. The inter-pulse delay T needs to be considerably longer than T_m and was set to a value of 5.4 μs for the measurements at 80 K. All other parameters were the same as listed above for a field-swept echo-detected experiment.

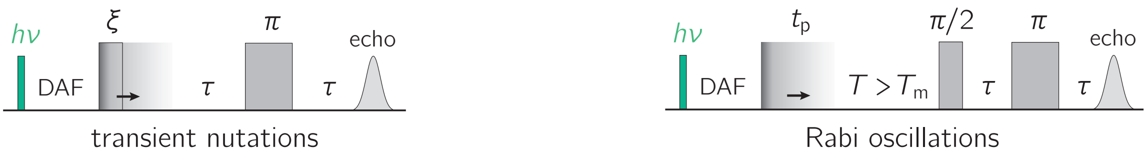

3.3 EPR characterisation of the radical and triplet precursors

The room temperature continuous wave (cw) EPR spectra of all three dyads, dissolved in toluene, are overlaid in Figure S11.

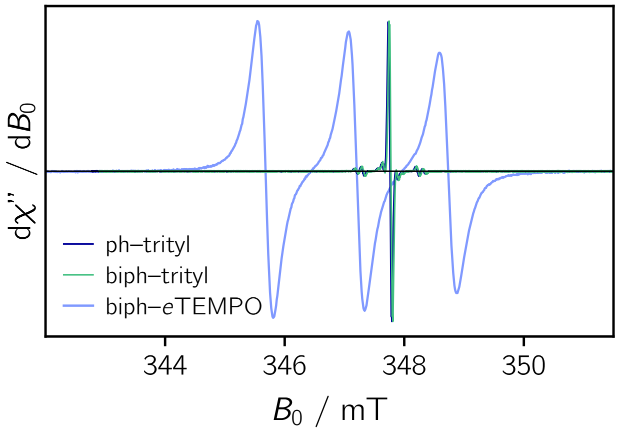

Figure S11: Comparison of the room temperature cw EPR data (field-modulated ≃ derivative shape) of all three investigated dyads.

As expected, the dark state spectra of the two PDI−trityl dyads are almost identical. It can be seen that the trityl radical is characterised by a very narrow EPR line with a peak-to-peak linewidth ΔB_{pp} of 0.056 mT, due to the absence of any magnetic nuclei in the vicinity of the unpaired electron spin. To the left and right of the central line, low-intensity satellite-peaks are observed that arise from hyperfine coupling to ^{13}C with natural abundance.[17]

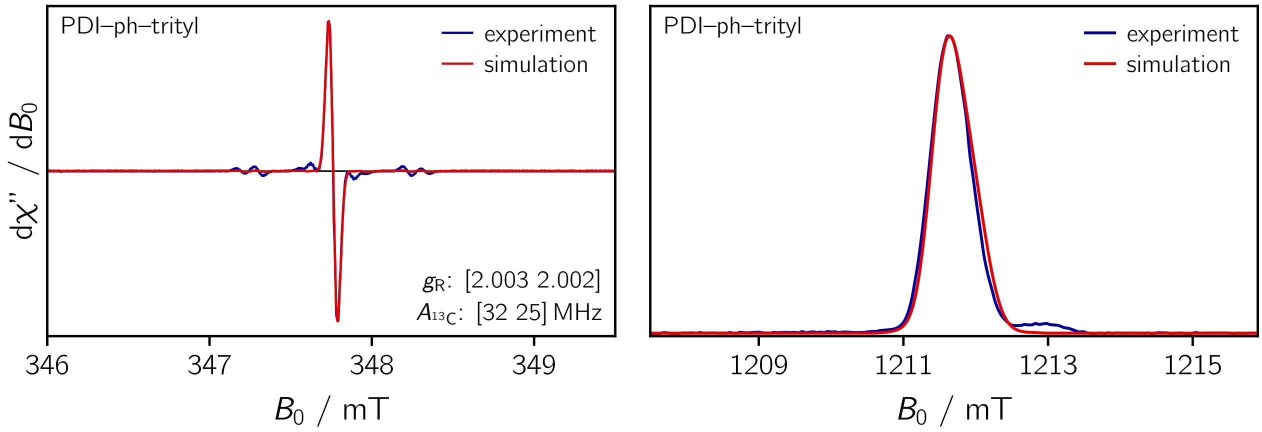

Figure S12: Determination of the **g** and **A** tensors of the trityl radical by simultaneous fitting of the X-band room temperature cw EPR spectrum of PDI−ph−trityl (*left*, field-modulated ≃ derivative shape) and the corresponding pulse Q-band spectrum recorded in frozen toluene solution at 80 K (*right*, direct detection).

The cw EPR spectrum of PDI−biph−*e*TEMPO exhibits the characteristics of a typical nitroxide spectrum. Three lines with a peak separation of ∼1.5 mT are observed, resulting from the coupling of the unpaired electron spin to the ^{14}N nucleus ($I = 1$) of the nitroxide group.

To determine the trityl **g** and **A** tensors, as needed for the simulation of the trEPR spectra of the

coupled dyads, cw EPR and pulse Q-band EPR data of PDI—ph—trityl were recorded and later fitted simultaneously. The data and fits are shown in Figure S12.

The triplet state parameters of the PDI chromophore were already determined elsewhere[18] and amount to $g_T = 2.00403$, $D_T = 970$ MHz, $E_T = -185$ MHz with initial relative zero-field populations of $P = [0.62\ 0.38\ 0]$ (from low to high energy).

3.4 Characterisation of the coupled systems by trEPR

Figure S13 shows the trEPR spectra of PDI—ph—trityl in toluene solution at different time delays. It can be seen that the shape of the signal varies significantly as a function of time. As expected, the quartet multiplet polarisation decays more rapidly than the net polarisation of the quartet state. In addition to the rather broad quartet state features centred at $g(\mathrm{Q}) = 2.0035$, two sharp peaks are observed in the centre of the trEPR spectra at early times after photoexcitation (up to ~1.5 µs). These additional features could potentially be assigned to the polarised trityl radical ground state (D_0, $g(\mathrm{D}_0) = 2.0025$) and the excited doublet state (D_1, $g(\mathrm{D}_1) = 2.0045$), based on the expected differences in their isotropic g values.[19] A similar observation is made for PDI—biph—trityl as shown in Figure S14.

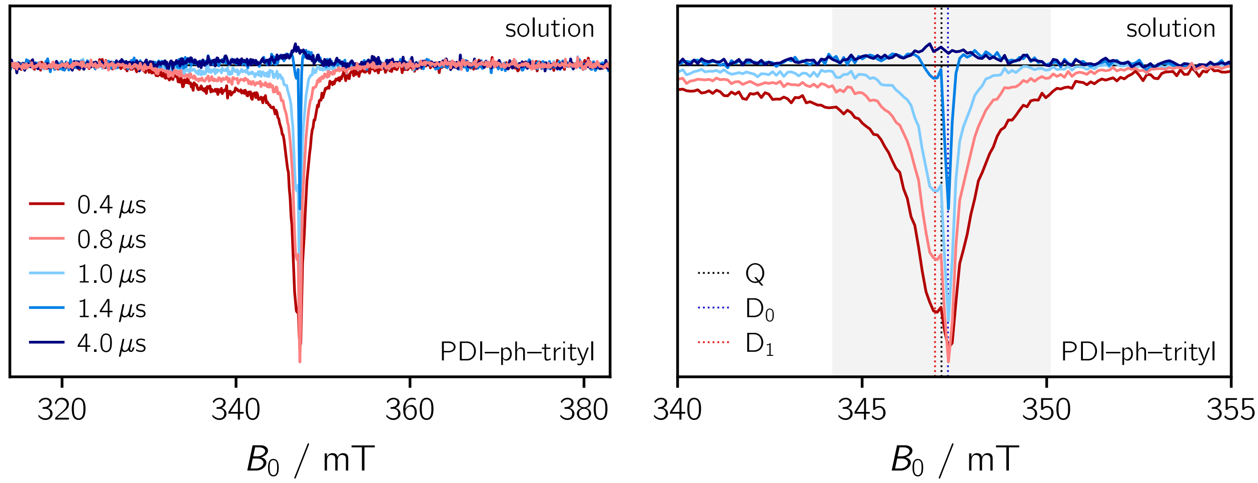

Figure S13: Room temperature trEPR spectra of PDI—ph—trityl in toluene solution at different time delays (as indicated) after laser excitation at 535 nm. The right panel shows a zoom into the data where the spectral positions corresponding to the isotropic g values of the quartet (Q) state, the polarised radical ground state (D_0), and the excited doublet state (D_1) are marked.

At the X-band, a g value difference of 0.001 only corresponds to a magnetic field shift of about 0.175 mT, while the same g value difference corresponds to a ΔB_0 of 0.6 mT at the Q-band. To clarify the presence of these different species further, room temperature solution trEPR measurements should be performed at higher microwave frequencies.

The trEPR spectra of PDI—ph—trityl and PDI—biph—trityl recorded in PMMA films at room temperature are compared in Figure S15. Since the spectral shape is found to change as a function of time after laser excitation, spectra at different time delays are shown in Figure S16.

For PDI—ph—trityl, we compared the spectral shape and trEPR signal decay under different conditions: (i) in frozen toluene solution at 80 K, (ii) in PMMA film at room temperature, and (iii) in toluene solution at room temperature. The results are shown in Figure S17.

It can be seen that the trEPR signal decays considerably faster in toluene solution at room temperature as compared to the corresponding signal obtained in a PMMA film. In addition, the width of the central

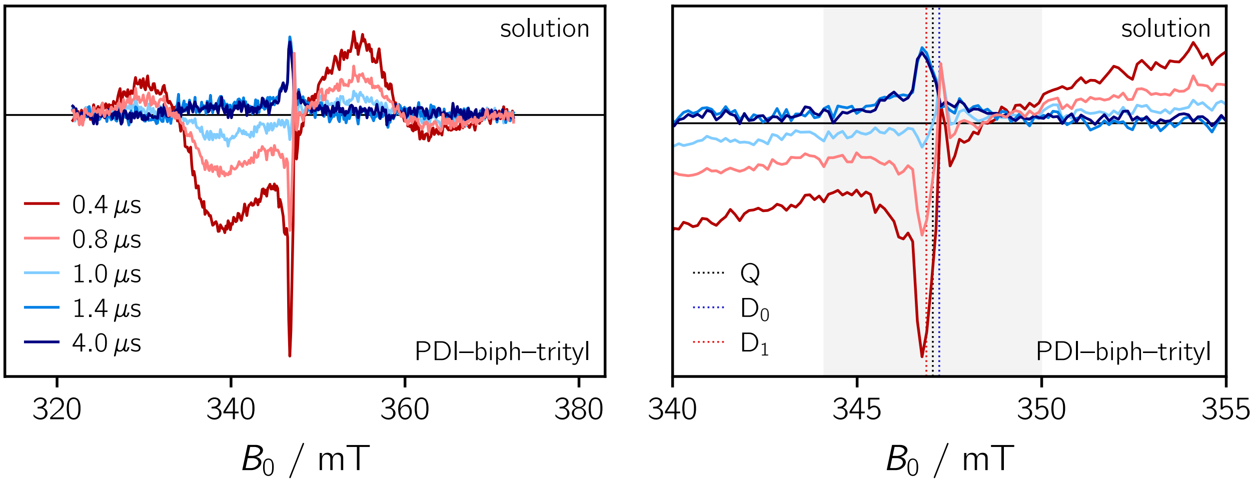

Figure S14: Room temperature trEPR spectra of PDI−biph−trityl in toluene solution at different time delays (as indicated) after laser excitation at 535 nm. The right panel shows a zoom into the data where the spectral positions corresponding to the isotropic *g* values of the quartet (Q) state, the polarised radical ground state (D_0), and the excited doublet state (D_1) are marked.

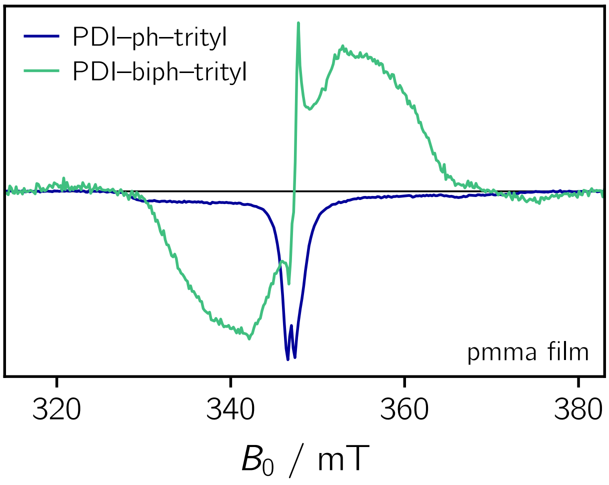

Figure S15: Comparison of the trEPR spectra recorded for PDI−ph−trityl and PDI−biph−trityl in PMMA films at room temperature.

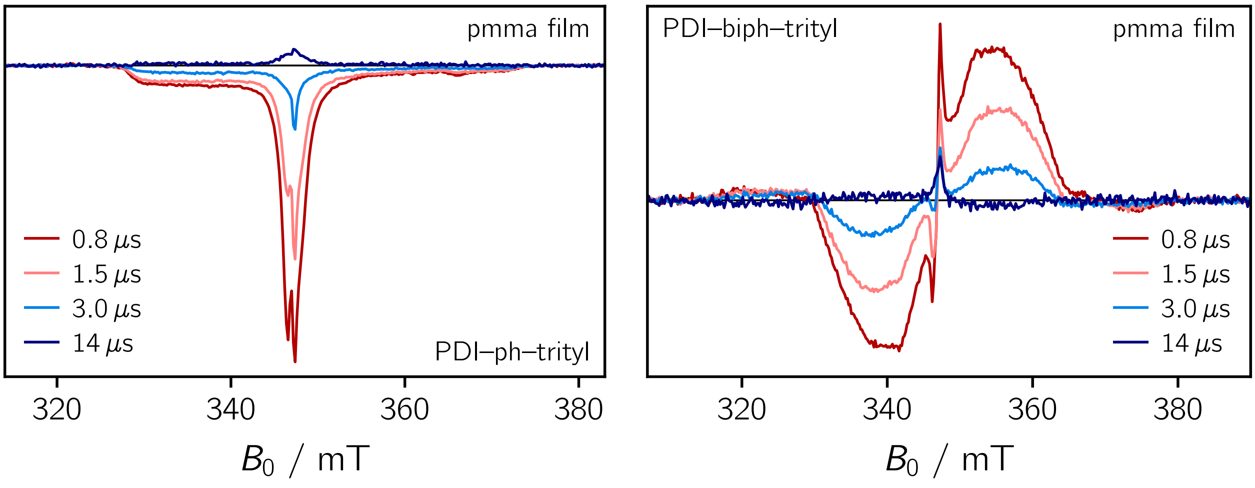

Figure S16: Room temperature trEPR spectra of PDI−ph−trityl (*left*) and PDI−biph−trityl (*right*) in PMMA films at different time delays after laser excitation (as indicated).

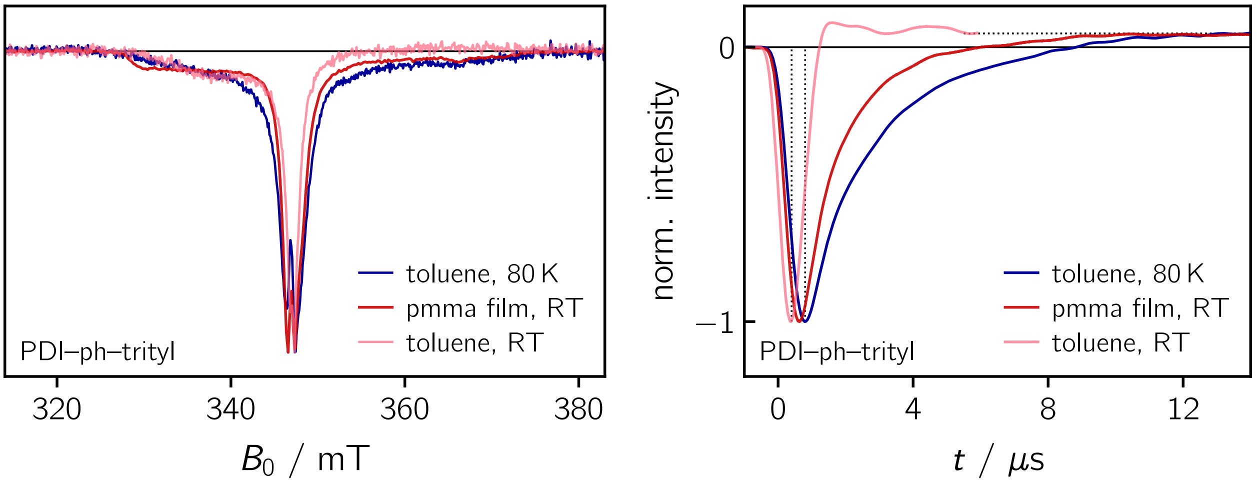

Figure S17: Comparison of the trEPR spectra at 0.8 µs after laser excitation (*left*) and the trEPR signal decay at a field position corresponding to the quartet $|+\frac{1}{2}\rangle \leftrightarrow |-\frac{1}{2}\rangle$ transition (*right*) of PDI−ph−trityl in frozen toluene solution with the corresponding data recorded at room temperature in a PMMA film and in toluene solution.

quartet feature is found to vary depending on the measurement condition. In all cases, the multiplet spin polarisation is found to have decayed after ∼10 µs. However, the signal does not decay to baseline level. Instead, an absorptive signal with an intensity of about 5 % of the initial signal intensity remains, attributed to the quartet net polarisation ($|+\frac{1}{2}\rangle \leftrightarrow |-\frac{1}{2}\rangle$ transition) based on its spectral position and width (see also Figure S16, *left*).

3.5 Simulation of the trEPR spectra

The room temperature trEPR spectra of PDI−ph−trityl and PDI−biph−trityl, shown in the main part, were simulated using the current developer version of EasySpin (v6.0.0-dev.50). [20,21] The function 'pepper' was used for the simulation of the powder spectra of the coupled spin systems and the simulation was carried out in the eigenbasis of the high-field states. During the fitting procedure, the magnetic parameters of the radical and chromophore precursor molecules ($\boldsymbol{g}_\mathrm{R}$, $\boldsymbol{g}_\mathrm{T}$, D_T) were held fixed, so that only the coupling parameters (D_TR, J_TR), linewidths, and the populations of the quartet spin states needed to be adapted. The results are summarised in Table S3.

The exchange interaction J_TR was assumed to be negative (corresponding to ferromagnetic coupling in EasySpin), in agreement with the exchange coupling calculations shown below (Subsection 4.4). Note that our exchange coupling calculations and the EasySpin software rely on a different definition of the exchange coupling Hamiltonian: in our quantum chemical calculations, a positive value of J_TR indicates ferromagnetic coupling (i.e. $E_\mathrm{Q} < E_\mathrm{D1}$).

Table S3: Simulation parameters for the coupled PDI–trityl systems.

	PDI−ph−trityl	PDI−biph−trityl
g_R	[2.003 2.002]	[2.003 2.002]
g_T	2.004	2.004
D_T / MHz	970	970
E_T / MHz	−185	−102
J_TR / MHz	−40000	−16600
D_TR / MHz	[148 148 −296]	[53 53 −106]
population ('eigen')		
$\mathrm{Q}_{+3/2}$	0.54	0.11
$\mathrm{Q}_{+1/2}$	0.07	0.46
$\mathrm{Q}_{-1/2}$	0	0.43
$\mathrm{Q}_{-3/2}$	0.39	0
HStrain / MHz	[109 29 16]	[52 12 6]
DStrain / MHz	[592 467]	[148 146]

3.6 Additional pulse EPR data

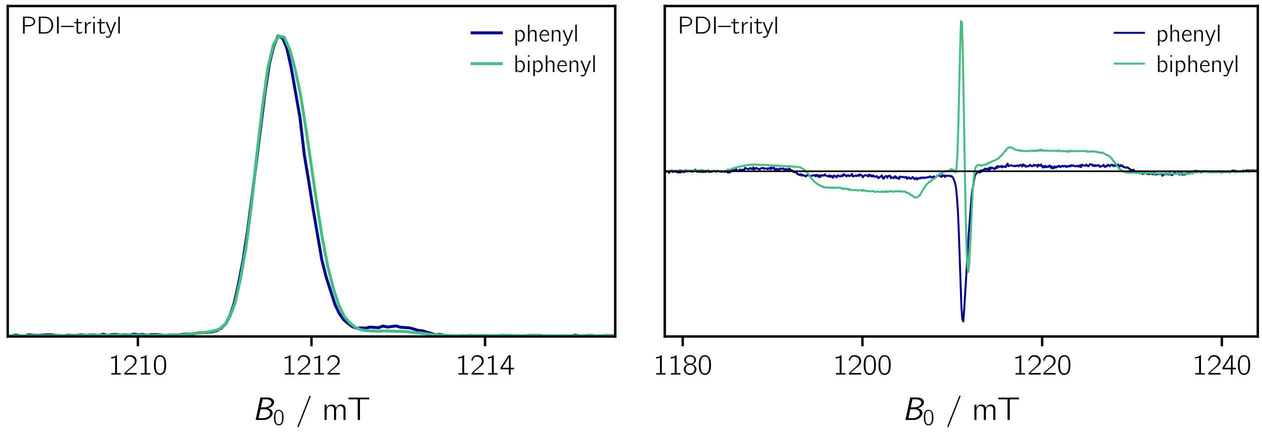

Figure S18: Comparison of the pulse EPR spectra of the PDI−trityl compounds recorded at the Q-band at 80 K in the dark (*left*) and after photoexcitation at 535 nm (*right*). The dark state background was subtracted from the light-induced spectra.

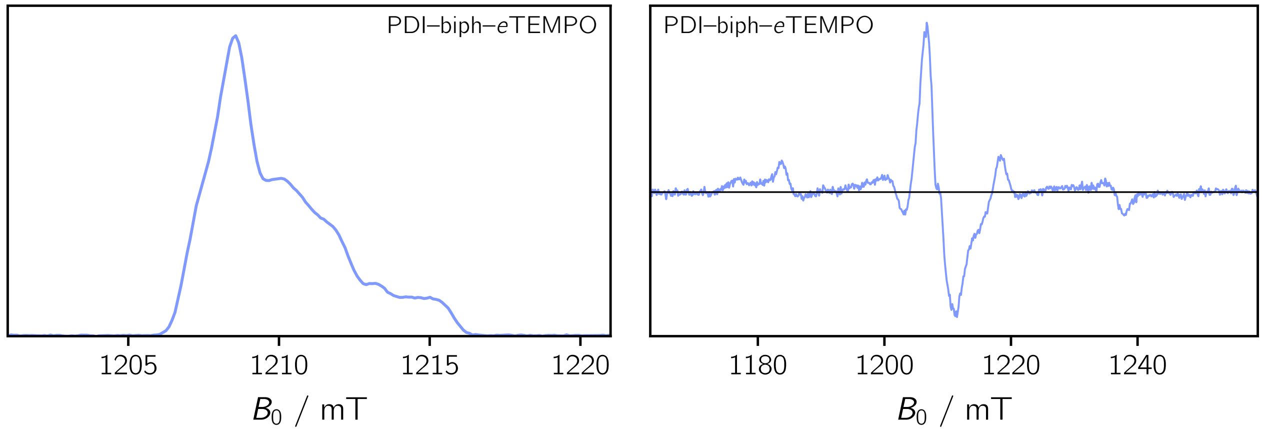

Figure S19: Pulse EPR spectra of PDI−biph−*e*TEMPO recorded at the Q-band at 80 K in the dark (*left*) and after photoexcitation at 535 nm (*right*). The dark state background was subtracted from the light-induced spectrum.

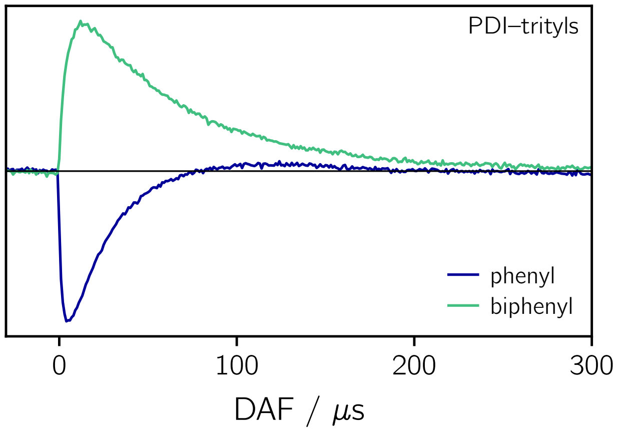

Figure S20: Comparison of the spin polarisation lifetime for PDI−ph−trityl and PDI−biph−trityl recorded at the Q-band at 80 K at field position corresponding to the central quartet line ($|-1/2\rangle \leftrightarrow |+1/2\rangle$ transition).

4 Quantum chemical calculations

All quantum chemical calculations were carried out using ORCA 5.0.3.[22]

4.1 Optimisation of the structures

The structures shown in Figure S21 and Figure S22 were optimised at the BP86/def2-SVP level of theory.[23] The calculations were accelerated with the RIJCOSX approximation using the def2/J auxiliary basis set.[24]

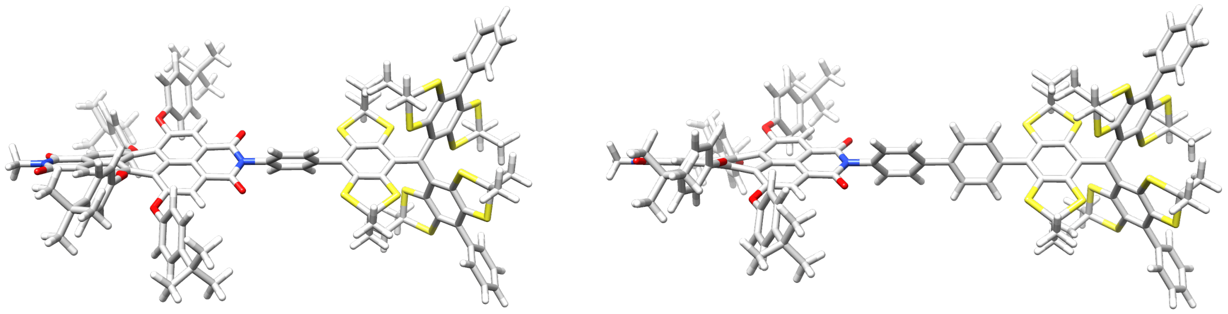

Figure S21: Structures of the PDI−trityl dyads calculated at the BP86/def2-SVP level of theory.

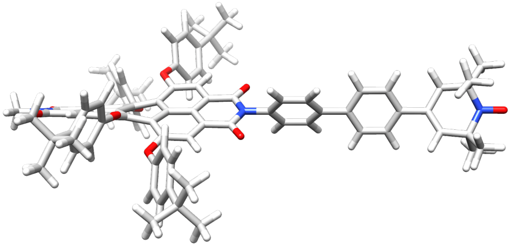

Figure S22: Structure of the PDI−biph−*e*TEMPO dyad calculated at the BP86/def2-SVP level of theory.

4.2 Triplet state energy of PDI

The adiabatic triplet state energy of PDI was calculated by considering the optimised structures of the singlet and triplet states which are shown in Figure S23. The optimisations were carried out at the M06/def2-TZVP level of theory.[23,25] The final energy of the triplet state was then calculated by a TDDFT calculation (M06/def2-TZVP level of theory) using the triplet state optimised geometry but starting from the restricted Kohn-Sham (RKS) singlet state. A value of $\Delta E = 1.30$ eV was obtained.

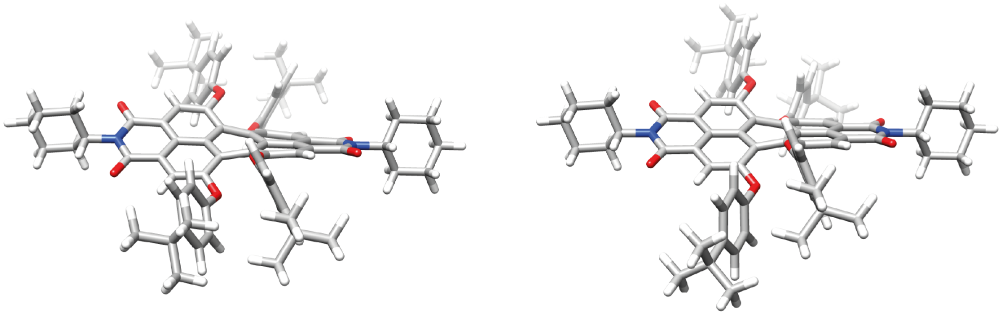

Figure S23: Structures of the S_0 and T_1 states of the PDI chromophore calculated at the M06/def2-TZVP level of theory.

A single TDDFT calculation using only the S_0 optimised structure usually leads to an overestimation of the triplet state energy, whereas the restricted open-shell Kohn-Sham (ROKS) energy of the triplet

state is too low. The protocol used in this calculation represents a compromise between the above-mentioned procedures and was benchmarked on a bare perylene structure where the experimental triplet state energy is known. Using this method, an excellent agreement between experiment and calculation could be obtained ($\Delta E = 1.57\,\text{eV}$, in both cases).

4.3 Excited state exchange couplings

The excited state exchange couplings were calculated at the QD-NEVPT2/def2-SVP level of theory. Here, the strongly contracted variant of the NEVPT2 method was used in its quasi-degenerate formulation.[26–28] Furthermore, the calculations were accelerated with the RIJCOSX approximation using the def2/J auxiliary basis set. The reference wavefunctions were obtained by a state-averaged CASSCF(3,3) calculation, whereby the quartet state and the trip-doublet state were equally weighted by 50 %. The active orbitals were selected either from a previous TDDFT calculation or a MP2 calculation. The molecules and their active orbitals are shown in Figure S24.

The values for J_{TR}, which are listed in Table S4, were calculated by using the QD-NEVPT2 energies of the quartet state and the trip-doublet state:

$$J_{\text{TR}} = \frac{2}{3}(E_{\text{D}_1} - E_{\text{Q}_0}). \tag{S8}$$

Table S4: Calculated values of J_{TR} for different PDI–based chromophore–radical systems.

Molecule	No.	J_{TR}
PDI–ph–trityl	**1**	0.0525 cm^{-1}
PDI–biph–trityl	**2**	0.00134 cm^{-1}
PDI–ph–*e*TEMPO	**3**	–0.00426 cm^{-1}
PDI–biph–*e*TEMPO	**4**	–0.00106 cm^{-1}
PDI–TEMPO	**5**	–0.00626 cm^{-1}

The individual exchange interactions J_{12} and J_{23}, between the radical SOMO orbital (spin 2) and the chromophore HOMO and LUMO orbitals (spins 1 and 3), contributing to J_{TR} according to[29]

$$J_{\text{TR}} = \frac{J_{12} + J_{23}}{2} \tag{S9}$$

were calculated by making use of the effective Hamiltonian theory. Here, the energies and wavefunctions of the quartet state, the trip–doublet state and the sing–doublet state are used to generate an effective Hamiltonian, whose eigenvalues are the energies of the three mentioned states. The three states are projected onto the subspace of neutral determinants, which allows for a one-to-one correspondence with the Heisenberg-Dirac-Van-Vleck Hamiltonian. The exact procedure is described in reference 29. The individual exchange interactions are given in Figure S24.

It should be noted here, that, for the two systems using trityl as the radical, the orbitals of the phenyl bridge may play an important role due to the highly delocalised nature of the radical. As a consequence, it is likely that the CAS(3,3) zeroth-order wavefunction is less well described in these two cases as compared to the other systems. One thus needs to be careful when comparing the results obtained for systems bearing different radicals. However, the results obtained for the two trityl-based systems should be comparable to each other.

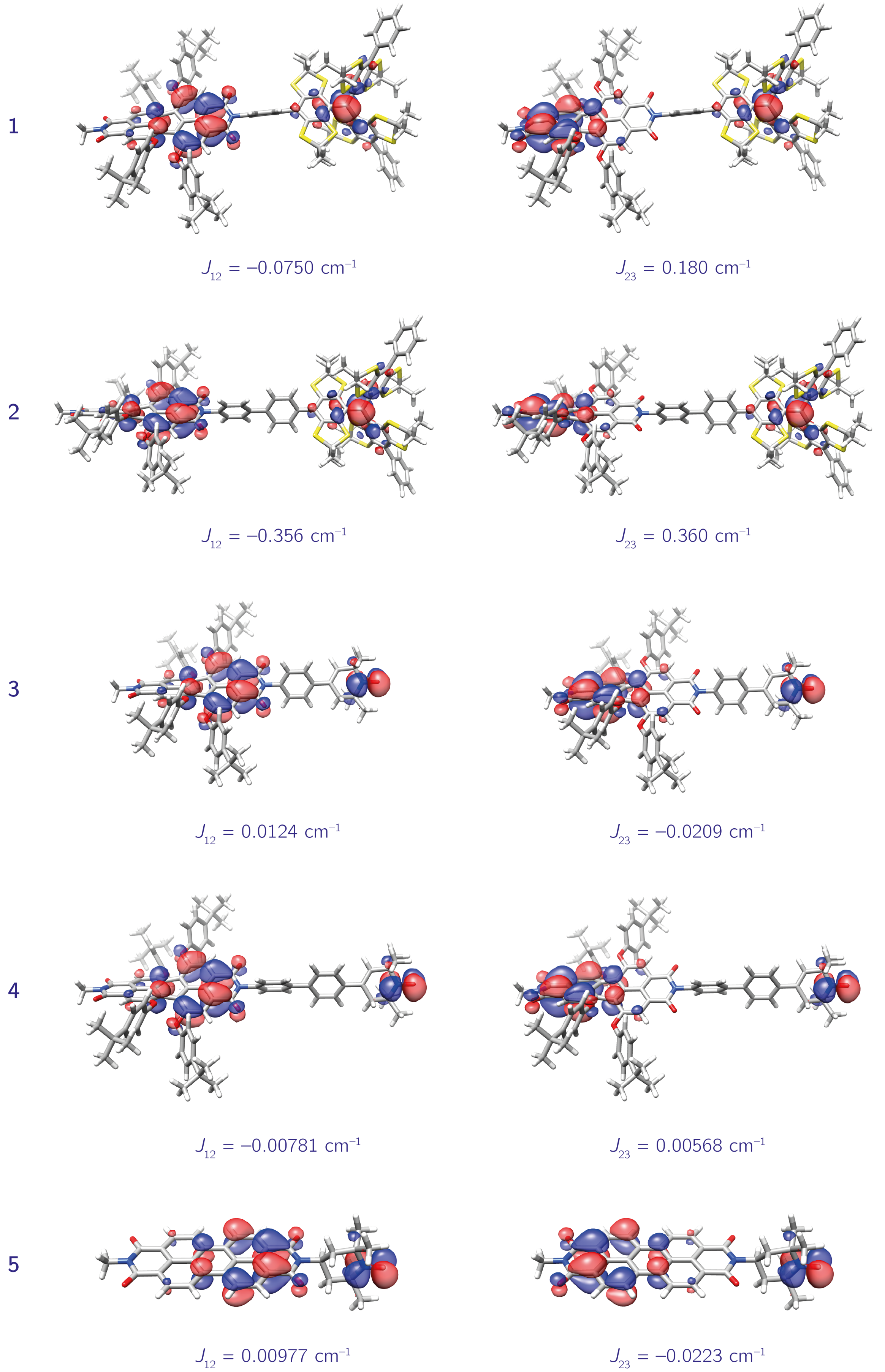

Figure S24: Active orbitals of different PDI–based chromophore–radical systems and the corresponding individual exchange interactions. The compound numbers correspond to those in the table above. Compounds **1** (PDI–ph–trityl), **2** (PDI–biph–trityl), and **4** (PDI–biph–*e*TEMPO) were investigated experimentally in this work. Compound **5** was discussed in references 30 and 2.

5 NMR spectra

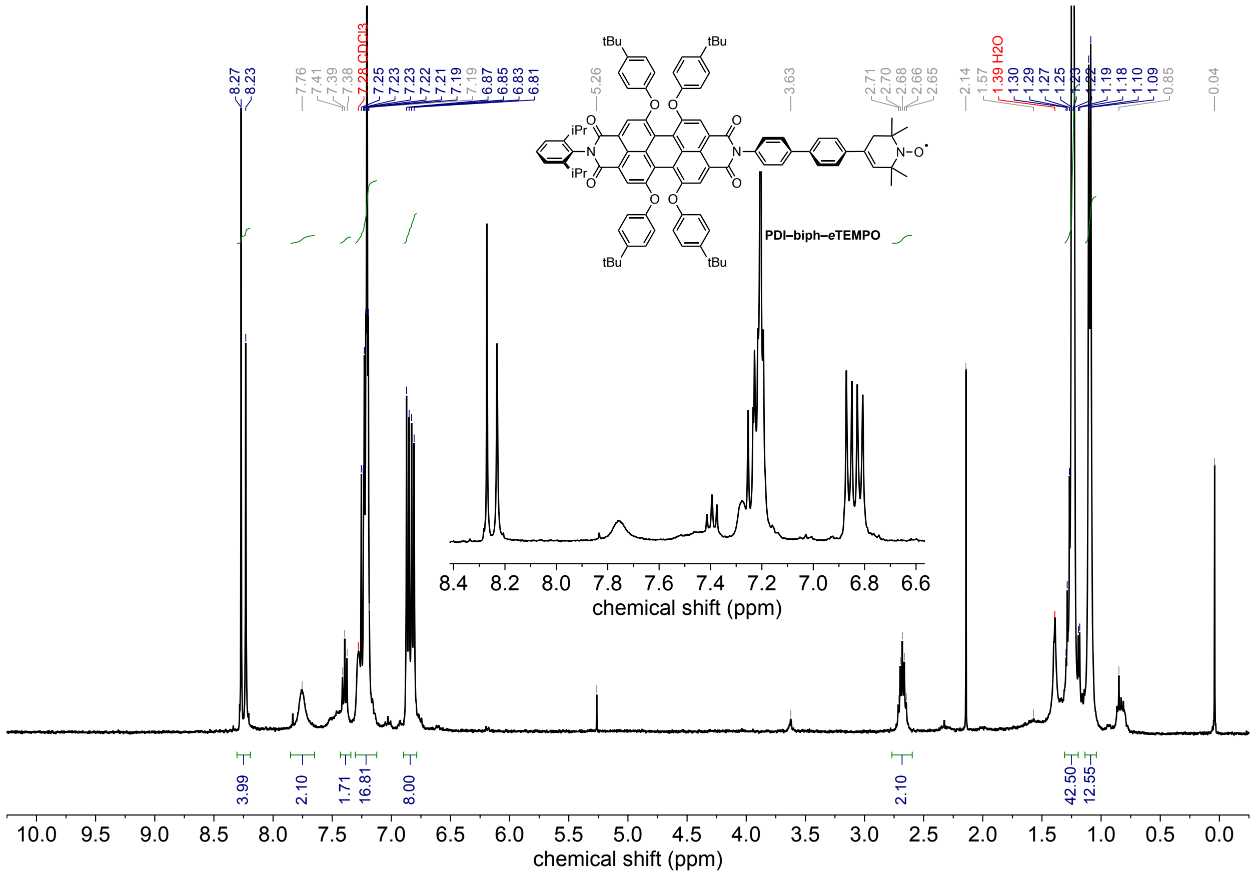

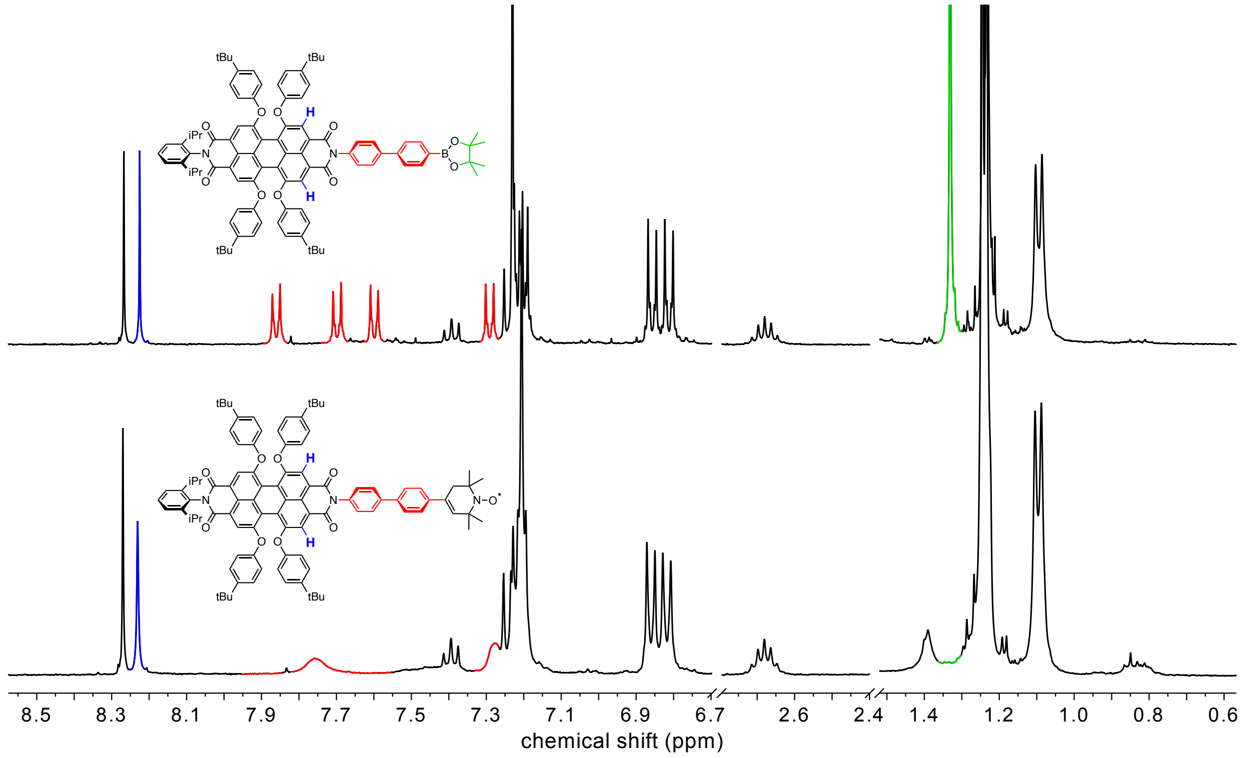

Figure S25: ¹H NMR spectrum of PDI−biph−*e*TEMPO (*top*) and comparison of the ¹H NMR spectra of PDI−biph−Bpin and PDI−biph−*e*TEMPO (*bottom*).

6 HRMS data

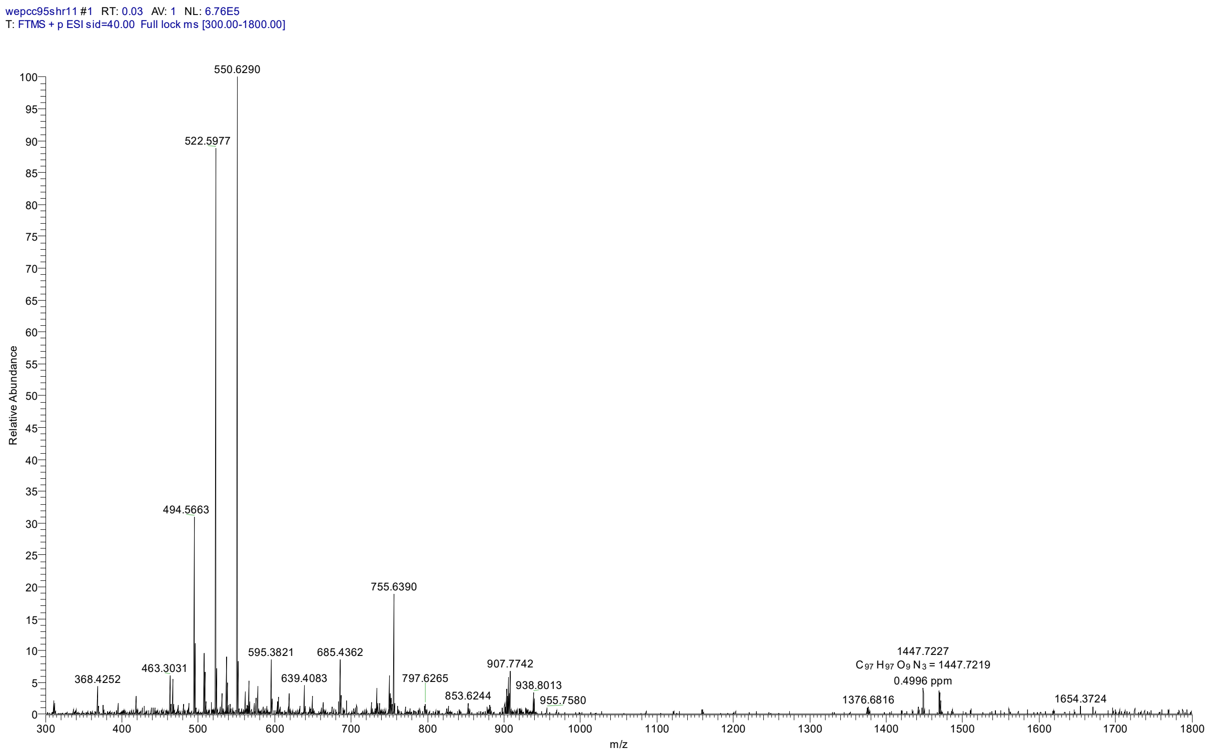

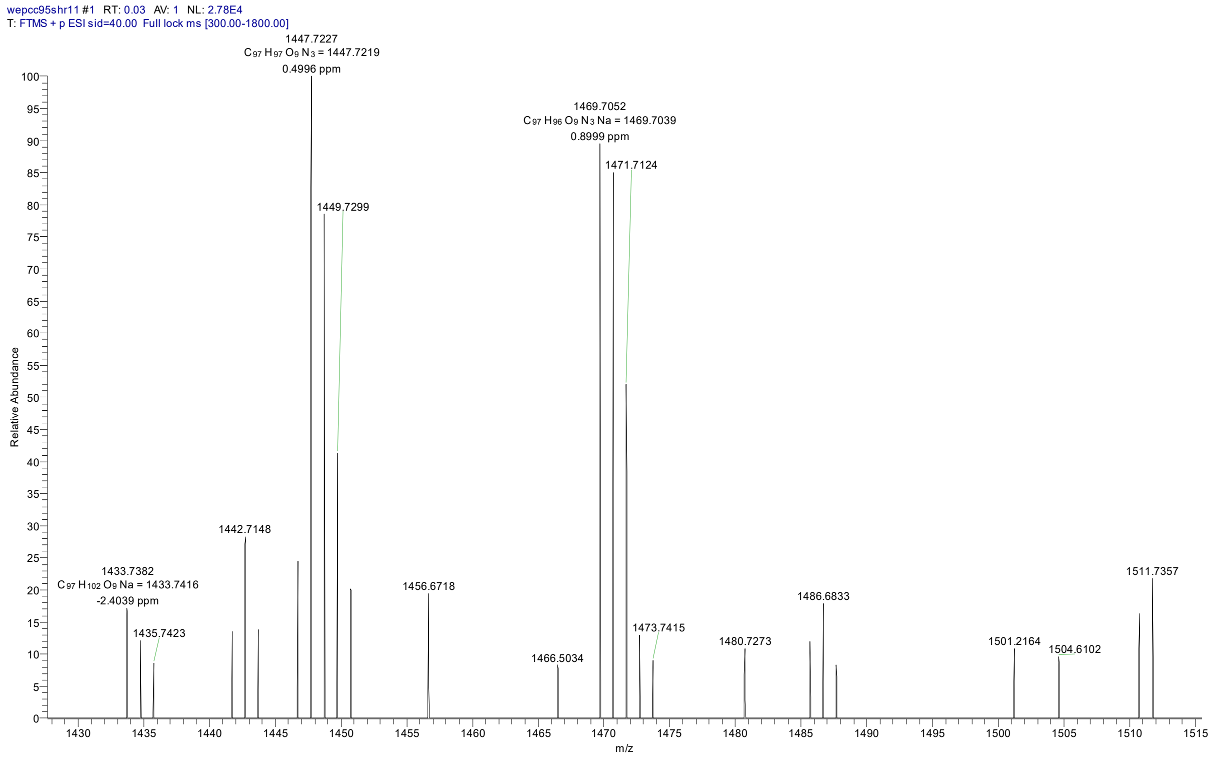

Figure S26: HRMS-ESI analysis for PDI−biph−*e*TEMPO.

References

[1] Mayländer, M.; Quintes, T.; Franz, M.; Allonas, X.; Vargas Jentzsch, A.; Richert, S. Distance dependence of enhanced intersystem crossing in BODIPY−nitroxide dyads. *Chem. Sci.* **2023**, *14*, 5361–5368.

[2] Mayländer, M.; Nolden, O.; Franz, M.; Chen, S.; Bancroft, L.; Qiu, Y.; Wasielewski, M. R.; Gilch, P.; Richert, S. Accessing the triplet state of perylenediimide by radical-enhanced intersystem crossing. *Chem. Sci.* **2022**, *13*, 6732–6743.

[3] Nolden, O.; Fleck, N.; Lorenzo, E. R.; Wasielewski, M. R.; Schiemann, O.; Gilch, P.; Richert, S. Excitation energy transfer and exchange-mediated quartet state formation in porphyrin-trityl systems. *Chem. Eur. J.* **2021**, *27*, 2683–2691.

[4] Hudson, J. M.; Hele, T. J. H.; Evans, E. W. Efficient light-emitting diodes from organic radicals with doublet emission. *J. Appl. Phys.* **2021**, *129*, 180901.

[5] Lakowicz, J. R. *Principles of fluorescence spectroscopy*; Springer: New York, 2006.

[6] Weller, A. Photoinduced electron transfer in solution: exciplex and radical ion pair formation free enthalpies and their solvent dependence. *Z. Phys. Chem.* **1982**, *133*, 93–98.

[7] Fleck, N.; Heubach, C. A.; Hett, T.; Haege, F. R.; Bawol, P. P.; Baltruschat, H.; Schiemann, O. SLIM: a short-linked, highly redox-stable trityl label for high-sensitivity in-cell EPR distance measurements. *Angew. Chem. Int. Ed.* **2020**, *132*, 9854–9859.

[8] Wu, Y.; Young, R. M.; Frasconi, M.; Schneebeli, S. T.; Spenst, P.; Gardner, D. M.; Brown, K. E.; Würthner, F.; Stoddart, J. F.; Wasielewski, M. R. Ultrafast photoinduced symmetry-breaking charge separation and electron sharing in perylenediimide molecular triangles. *J. Am. Chem. Soc.* **2015**, *137*, 13236–13239.

[9] Wohlfarth, C. In *Landolt-Börnstein, New Series*; Lechner, M. D., Ed.; Springer: Berlin, 2008.

[10] Laimgruber, S.; Schachenmayr, H.; Schmidt, B.; Zinth, W.; Gilch, P. A femtosecond stimulated raman spectrograph for the near ultraviolet. *Appl. Phys. B* **2006**, *85*, 557–564.

[11] Laimgruber, S.; Schmierer, T.; Gilch, P.; Kiewisch, K.; Neugebauer, J. The ketene intermediate in the photochemistry of *ortho*-nitrobenzaldehyde. *Phys. Chem. Chem. Phys.* **2008**, *10*, 3872–3882.

[12] Fröbel, S.; Buschhaus, L.; Villnow, T.; Weingart, O.; Gilch, P. The photoformation of a phthalide: a ketene intermediate traced by FSRS. *Phys. Chem. Chem. Phys.* **2015**, *17*, 376–386.

[13] Reiffers, A.; Torres Ziegenbein, C.; Schubert, L.; Diekmann, J.; Thom, K. A.; Kühnemuth, R.; Griesbeck, A.; Weingart, O.; Gilch, P. On the large apparent Stokes shift of phthalimides. *Phys. Chem. Chem. Phys.* **2019**, *21*, 4839–4853.

[14] Lorenc, M.; Ziolek, M.; Naskrecki, R.; Karolczak, J.; Kubicki, J.; Maciejewski, A. Artifacts in femtosecond transient absorption spectroscopy. *Appl. Phys. B* **2002**, *74*, 19–27.

[15] van Stokkum, I. H. M.; Larsen, D. S. L.; van Grondelle, R. Global and target analysis of time-resolved spectra. *Biochim. Biophys. Acta Bioenerg.* **2004**, *1657*, 82–104.

[16] Herb, K.; Tschaggelar, R.; Denninger, G.; Jeschke, G. Double resonance calibration of g factor standards: carbon fibers as a high precision standard. *J. Magn. Reson.* **2018**, *289*, 100–106.

[17] Fleck, N.; Hett, T.; Brode, J.; Meyer, A.; Richert, S.; Schiemann, O. C-C cross-coupling reactions of trityl radicals: spin density delocalization, exchange coupling and a spin label. *J. Org. Chem.* **2019**, *84*, 3293–3303.

[18] Mayländer, M.; Thielert, P.; Quintes, T.; Vargas Jentzsch, A.; Richert, S. Room temperature electron spin coherence in photogenerated molecular spin qubit candidates. *J. Am. Chem. Soc.* **2023**, *145*, 14064–14069.

[19] Bencini, A.; Gatteschi, D. *EPR of exchange coupled systems*; Dover Publications: New York, 2012.

[20] Stoll, S.; Schweiger, A. EasySpin, a comprehensive software package for spectral simulation and analysis in EPR. *J. Magn. Reson.* **2006**, *178*, 42–55.

[21] Tait, C. E.; Krzyaniak, M. D.; Stoll, S. Computational tools for the simulation and analysis of spin-polarized EPR spectra. *J. Magn. Reson.* **2023**, *349*, 107410.

[22] Neese, F.; Wennmohs, F.; Becker, U.; Riplinger, C. The ORCA quantum chemistry program package. *J. Chem. Phys.* **2020**, *152*, 224108.

[23] Weigend, F.; Ahlrichs, R. Balanced basis sets of split valence, triple zeta valence and quadruple zeta valence quality for H to Rn: Design and assessment of accuracy. *Phys. Chem. Chem. Phys.* **2005**, *7*, 3297.

[24] Neese, F.; Wennmohs, F.; Hansen, A.; Becker, U. Efficient, approximate and parallel Hartree–Fock and hybrid DFT calculations. A 'chain-of-spheres' algorithm for the Hartree–Fock exchange. *Chem. Phys.* **2009**, *356*, 98–109.

[25] Zhao, Y.; Truhlar, D. G. The M06 suite of density functionals for main group thermochemistry, thermochemical kinetics, noncovalent interactions, excited states, and transition elements: two new functionals and systematic testing of four M06-class functionals and 12 other functionals. *Theor. Chem. Acc.* **2008**, *120*, 215–241.

[26] Angeli, C.; Cimiraglia, R.; Evangelisti, S.; Leininger, T.; Malrieu, J.-P. Introduction of n-electron valence states for multireference perturbation theory. *J. Chem. Phys.* **2001**, *114*, 10252–10264.

[27] Angeli, C.; Cimiraglia, R.; Malrieu, J.-P. N-electron valence state perturbation theory: a fast implementation of the strongly contracted variant. *Chem. Phys. Lett.* **2001**, *350*, 297–305.

[28] Angeli, C.; Borini, S.; Cestari, M.; Cimiraglia, R. A quasidegenerate formulation of the second order *n*-electron valence state perturbation theory approach. *J. Chem. Phys.* **2004**, *121*, 4043–4049.

[29] Franz, M.; Neese, F.; Richert, S. Calculation of exchange couplings in the electronically excited state of molecular three-spin systems. *Chem. Sci.* **2022**, *13*, 12358–12366.

[30] Mayländer, M.; Chen, S.; Lorenzo, E. R.; Wasielewski, M. R.; Richert, S. Exploring photogenerated molecular quartet states as spin qubits and qudits. *J. Am. Chem. Soc.* **2021**, *143*, 7050–7058.
